# New Meroterpenoid and Isocoumarins from the Fungus *Talaromyces amestolkiae* MST1-15 Collected from Coal Area

**DOI:** 10.3390/molecules27238223

**Published:** 2022-11-25

**Authors:** Kai-Yu Li, Qin-Feng Zhu, Jun-Li Ao, Fu-Rui Wang, Xing-Mei Long, Shang-Gao Liao, Guo-Bo Xu

**Affiliations:** 1 State Key Laboratory of Functions and Applications of Medicinal Plants, School of Pharmacy, Guizhou Medical University, Guian New District, Guiyang 550025, China; 2 Engineering Research Center for the Development and Application of Ethnic Medicine and TCM, Ministry of Education & Guizhou Provincial Key Laboratory of Pharmaceutics, Guiyang 550004, China

**Keywords:** *Talaromyces amestolkiae*, meroterpenoid, isocoumarin, antibacterial activity, *Stenotrophomonas maltophilia*

## Abstract

Three new compounds including a meroterpenoid (**1**) and two isocoumarins (**8** and **9**), together with thirteen known compounds (**2–7**, **10–16**) were isolated from the metabolites of *Talaromyces amestolkiae* MST1-15. Their structures were identified by a combination of spectroscopic analysis. The absolute configuration of compound **1** was elucidated on the basis of experimental and electronic circular dichroism calculation, and compounds **8** and **9** were determined by Mo_2_(OAc)_4_-induced circular dichroism experiments. Compounds **7–16** showed weak antibacterial activities against *Stenotrophomonas maltophilia* with MIC values ranging from 128 to 512 μg/mL (MICs of ceftriaxone sodium and levofloxacin were 128 and 0.25 μg/mL, respectively).

## 1. Introduction

The genus *Talaromyces* was initially established by Benjamin and over 71 species have been reported [1,2]. Secondary metabolites from *Talaromyces* are rich in structural diversity including anthraquinones, alkaloids, terpenes, and azaphilones, and had a wide range of bioactivities, such as MAO-inhibitory, nematicidal, cytotoxic, antiviral, and anti-inflammatory activities [3,4,5]. *Talaromyces amestolkiae* belongs to the genus *Talaromyces*, species of which are found to widely inhabit plants, soil, sponges, and foods [6]. This fungus is increasingly attracting attention for its ability to produce high levels of xylanases, cellulases, active compounds, and natural colorants with potential applications in the fields of industry, medicine, and food [7,8]. Recently, a series of different structural types of compounds including meroterpenoids, isocoumarins, and benzofurans were isolated as metabolites of *T. amestolkiae* for activity screening [5,6,9,10].

Extremophiles have been proven to be a promising source of bioactive compounds with diverse structures since their first discovery in the mid-20th century. Most fungi that inhabit extreme environments are categorized as Ascomycota covering a range of genera, including *Acremonium*, *Alternaria*, *Aspergillus*, *Cladosporium*, and *Penicillium*, and have been demonstrated to produce numerous worthwhile compounds with antimicrobial, anticancer, and/or antidiabetic activities [11,12,13]. Coal areas are deemed as multi-extreme environments with high metal contents and/or extreme pH, in which fungi survive through producing extremolytes, extremozymes, as well as small useful molecules to cope with the extreme environments [14]. In our ongoing research on the discovery of bioactive compounds from fungi, a chemical study of the secondary metabolites of *T. amestolkiae* MST1-15 collected from the Xingren coal area was carried out. Sixteen compounds (**1**–**16**, Figure S1) including new meroterpenoid (**1**) and isocoumarins (**8** and **9**) were isolated. Compounds **7**–**16** showed weak antibacterial activities against *Stenotrophomonas maltophilia* with MIC values ranging from 128 to 512 μg/mL. Here, the isolation, structure elucidation, and antibacterial activities of these compounds are described.

## 2. Results and Discussion

### Structures Elucidation

**Figure 1:**
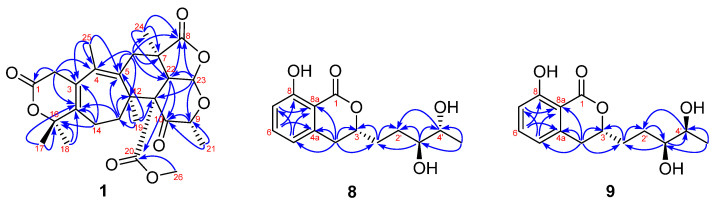
The Key HMBC correlations of compounds **1**, **8**, and **9**.

**Figure 2:**
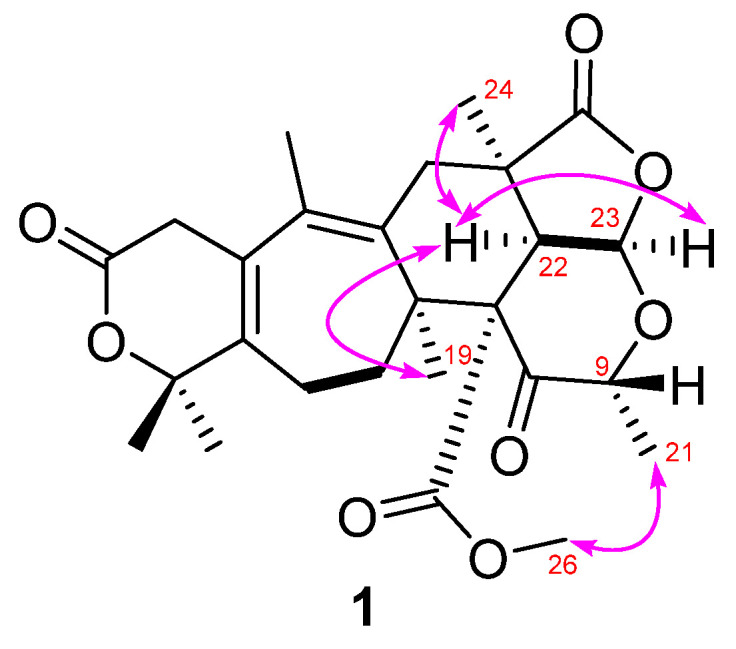
The Key NOESY (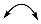) and ^1^H-^1^H COSY (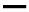) correlations of **1**.

Compound **1** was obtained as a white amorphous powder. The molecular formula was determined as C_26_H_32_O_8_ according to the HR ESIMS data with a [M−H]^−^ ion peak at *m/z* 471.2025 (calcd for 471.2024), implying 11 degrees of unsaturation. The ^1^H NMR spectrum of **1** exhibited five singlet methyl signals (δ_H_ 1.77, 1.57, 1.48, 1.33, and 1.14), a methoxyl peak (δ_H_ 3.80, s), and one doublet methyl peak (δ_H_ 1.42 (3H, d, *J* = 6.8 Hz)). With the aid of HSQC, ^13^C NMR data (Table 1) analysis revealed the presence of seven methyls (δ_C_ 53.2, 27.9, 27.4, 27.3, 26.5, 18.3, and 17.7), four methylenes (δ_C_ 49.1, 34.0, 31.6, and 26.7), three methines (98.5, 72.3 and 46.0), four sp^3^ quaternary carbons (δ_C_ 85.6, 63.2, 44.1, and 41.0), three ester carbonyls (δ_C_ 179.8, 169.4, and 168.2), one keto carbonyl (δ_C_ 209.4), four olefinic carbons (δ_C_ 137.0, 136.3, 132.8, and 126.9). The aforementioned data suggested that compound **1** was a meroterpenoid [6,15], and these spectroscopic features were very similar to those of berkeleyacetal A [16]; the major difference being the replacement of the olefinic methine (C*H*-14) by an additional olefinic quaternary carbon in **1**, suggesting **1** to be an olefinic positional isomer of berkeleyacetal A. Detailed 2D NMR (^1^H-^1^H COSY, HSQC, HMBC, and NOESY) data analysis (Figure 1 and Figure 2) further confirmed this deduction and figured out the position of the olefinic bonds. In particular, a Δ^3,15^ double bond was established by the HMBC correlations from H-2 (δ_H_ 3.32) to C-1 (δ_C_ 169.4), C-3 (δ_C_ 126.9), and C-15 (δ_C_ 136.3), while a Δ^4,5^ double bond was revealed by the HMBC correlations from H_3_-25 (δ_H_ 1.77) to C-3, C-4 (δ_C_ 132.8), and C-5 (δ_C_ 137.0) (Figure 1). The NOESY correlations of H-22 (δ_H_ 3.69) with H_3_-19 (δ_H_ 1.14) and H_3_-24 (δ_H_ 1.33) indicated that CH_3_-19, H-22, and CH_3_-24 are co-facially oriented and was assigned α orientation. In addition, a strong NOESY correlation observed for H-22 and H-23 (δ_H_ 6.09) suggested the *cis*-fusion of the furan and pyran rings. These observations showed that both berkeleyacetal A and **1** shared the same stereochemistry at almost all stereocenters. Moreover, the NOESY correlation of H_3_-26 (δ_H_ 3.80) with H_3_-21 (δ_H_ 1.42) indicated that both CH_3_-26 and CH_3_-21 were also on the same side of the hexa-ring. However, due to the absence of NOESY correlations between H_3_-21 and H-23, H-22, or H_3_-19, the configurations of C-9 and C-11 remained unclear.

**Figure 3:**
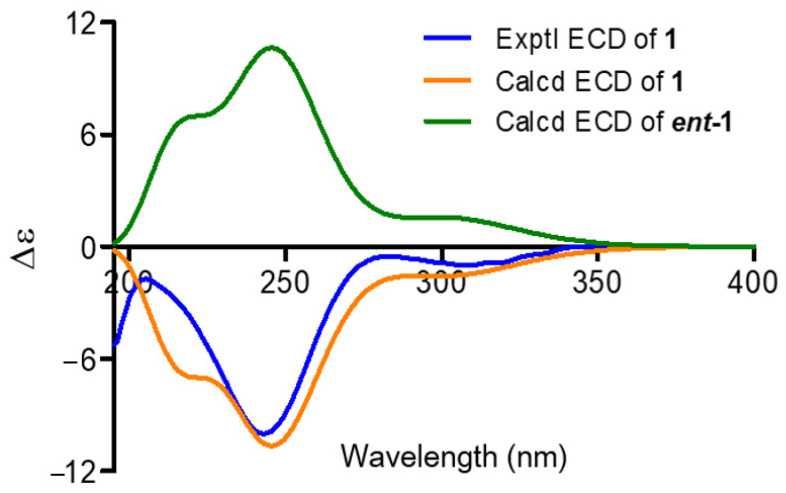
Experimental and calculated ECD spectra of **1**.

To further establish the relative configuration of C-9 and C-11, the chemical shifts of four isomers (**1a**–**1d**, Figure S35) were predicted at the B3LYP/6-311+G(d,p) with the PCM solvent model. The calculated chemical shifts of isomer **1a** were assigned to be in agreement with the experimental values according to the DP4+ analyses (Table S1). The absolute configuration of compound **1** was finally determined by a comparison of its experimental ECD spectrum with that calculated for the proposed structure by quantum chemical TDDFT. The predicted ECD spectrum of 7*R*, 9*R*, 11*S*, 12*S*, 22*R*, and 23*R* was in good agreement with that of the experimental one that showed a negative Cotton effect around 243 nm (Figure 3). Therefore, the absolute configuration of **1** was established as (7*R*, 9*R*, 11*S*, 12*S*, 22*R*, and 23*R*) and was named amestolkolide E.

Compound **8** was obtained as a white amorphous powder with the molecular formula of C_14_H_18_O_5_ based on its HR-ESI MS peak at *m*/*z* 265.1081 [M − H]^−^. Analysis of ^1^H and ^13^C NMR spectra suggested that compound **8** was an isocoumarin. The ^1^H NMR spectrum exhibited signals for three aromatic protons at δ_H_ 7.42 (1H, dd, *J* = 8.3, 7.3 Hz), 6.81 (1H, d, *J* = 8.3 Hz), and 6.76 (1H, d, *J* = 7.3 Hz), suggesting a 1,2,3-trisubstituted benzene motif in the structure, three oxygenated methines (δ_H_ 4.63, 3.55, and 3.35), one methyl (δ_H_ 1.16, d, *J* = 6.3 Hz), and three methylenes (δ_H_ 2.94 (1H, dd, *J* = 16.2, 11.2 Hz), 3.01 (1H, dd, *J* = 16.2, 3.0 Hz); 2.05 (1H, m), 1.77 (2H, m), 1.57 (1H, m)). With the aid of HSQC, the ^13^C NMR spectrum revealed 14 carbon signals for six aromatic carbons (δ_C_ 163.2, 141.6, 137.4, 116.7, 119.3, and 109.5), one ester carboxyl (δ_C_ 171.5), one methyl (δ_C_ 18.8), three methylenes (δ_C_ 33.6, 32.3, and 28.9), and three sp^3^ methines (δ_C_ 81.2, 76.2, and 71.8). All these spectroscopic data were very similar to those of aspergillumarin B [17] except for those for an extra oxymethine (δ_H_/δ_C_ 3.35/76.2 or 3.55/71.8) in **8**. The isochroman fragment was further confirmed by HMBC (Figure 1). The C_1′_-C_5′_ located at C-3 was demonstrated by HMBC correlations from H-1′ to C-3 and C-2′, from H-3′ to C-1′, C-2′, and C-4′, and from H-5′ to C-3′ and C-4′. Therefore, the planar structure of compound **8** was identified as 3,4-dihydroxypentyl-8-hydroxyisochroman-1-one. The ^1^H and ^13^C NMR data of compound **9** were nearly the same as compound **8** (Table 2) suggesting **9** to be an isomer of **8**, which was further confirmed by the HMBC correlation analysis (Figure 1).

**Scheme 1:**
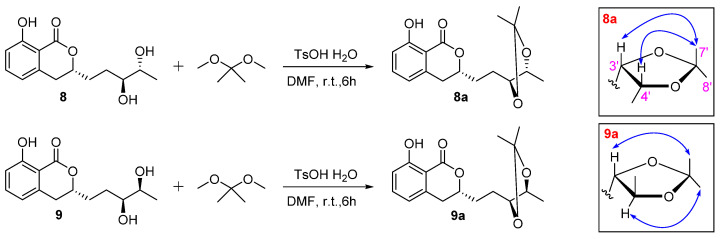
Synthesis of di-*O*-isopropylidene derivatives **8a** and **9a**, and NOESY correlations (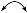) of **8a** and **9a**.

**Figure 4:**
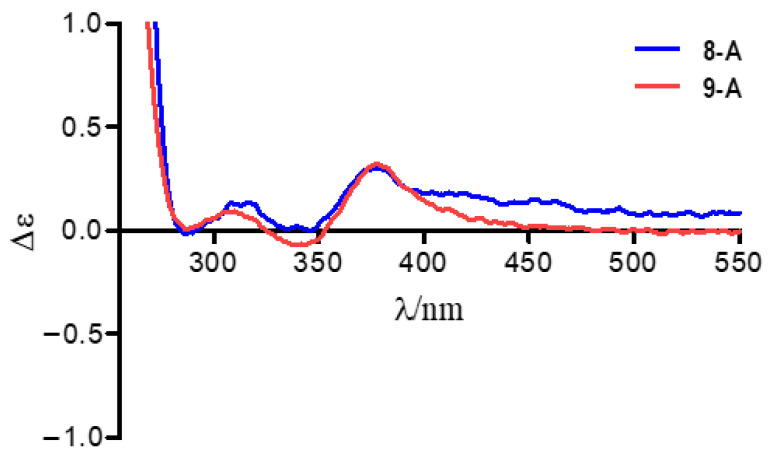
ICD spectra of compounds **8-A** (for **8**) and **9-A** (for **9**) and Mo_2_(OAc)_4_ in DMSO.

**Figure 5:**
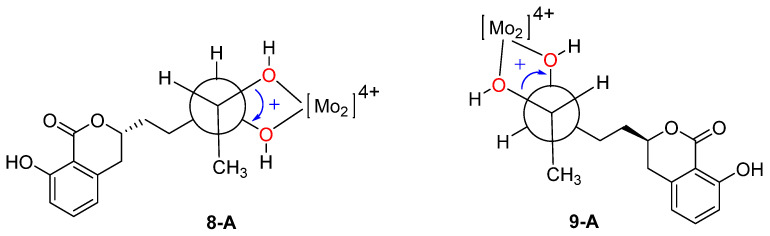
Favored conformers **8-A** and **9-A** for compounds **8** and **9** reacted with Mo_2_(OAc)_4_.

**Figure 6:**
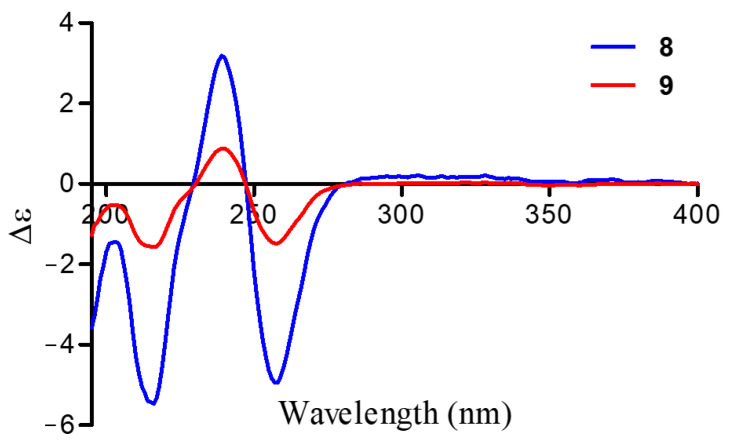
Experimental ECD spectra of **8** and **9**.

Since the relative stereochemistry of the vicinal hydroxyl groups in compounds **8** and **9** cannot be determined by their coupling constants, compounds **8** and **9** were reacted with 2,2-dimethoxypropane to yield their di-*O*-isopropylidene derivatives **8a** and **9a** (Scheme 1) [18]. ROESY experiment displayed correlations of H_3_-7′ with H-3′ and H-4′, suggesting CH_3_-7′, H-3′, and H-4′ to be cofacial in **8a**. An *erythro* configuration was therefore deduced for the vicinal diol **8**. Similarly, a *threo* configuration was established for **9** by the ROESY correlations of H_3_-7′/H-3′ and H_3_-8′/H-4′in **9a**. Subsequently, the dimolybdenum tetraacetate [Mo_2_(OAc)_4_]-induced CD (ICD) spectra were further applied to determine the absolute configurations for **8** and **9**. The ICD spectrum of **8** with [Mo_2_(OAc)_4_] in DMSO showed a positive Cotton effect at 317 nm (Figure 4), corresponding to a positive chirality for the O-C-C-O moiety according to Snatzke’s rule [19]; thus, an absolute configuration of *3*′*S* and *4*′*R* was figured out for conformation **8-A** (Figure 5) and then for **8**. Similarly, a positive Cotton effect at 310 nm in the ICD spectrum of **9-A** revealed the absolute stereochemistry of **9** to be 3′*S* and 4′*S*. The remaining absolute configurations of C-3 for **8** and **9** were determined by ECD, where the negative Cotton effects at 258 and 257nm (Figure 6) were consistent with a 3*R* in **8** and **9**, respectively [20].

**Figure 7:**
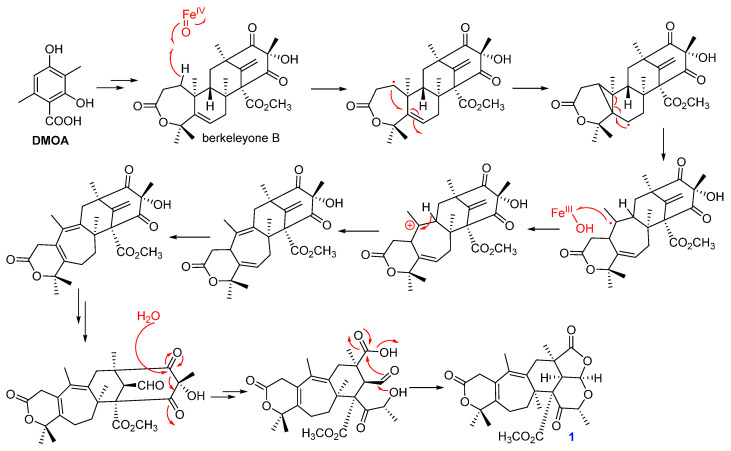
Plausible biosynthetic pathway of compound **1**.

Thirteen known compounds were isolated and identified as berkeleyacetals A (**2**) and B (**3**) [16], amestolkolide B (**4**) [6], chrysogenolide C (**5**) [21], purpurogenolides C (**6**) and E (**7**) [11], peniciisocoumarins F (**10**) and H (**15**) [22], 3*R*-((*R*)-4,5-dihydroxypentyl)-8-hydroxyisochroman-1-one (**11**) [5], aspergillumarins A (**13**) [17] and B(**12**) [23], 3*R*-8-methoxy-3-(4-oxopentyl) isochroman-1-one (**14**) [20], and penicimarin B (**16**) [23] by comparison of their optical values and spectroscopic data with those reported. The genera *Talaromyces*, a sexual state of the *Penicillium* species, can be found in a wide range of habitat conditions including extreme environments. At present, over fifty compounds of meroterpenoid, isocoumarin, azaphilone, and rare chemical skeletons (e.g., amestolkin) have been isolated from the metabolites of *T. amestolkiae* [5,6,9,10,24]. Meroterpenoids are the representative skeleton compounds isolated from the genus *Penicillium* and *Talaromyces*, biosynthesized from 3,5-dimethylorsellinic acid (DMOA) via a serial reaction such as epoxide-initiated cyclization, oxidative transformation, retro-claisen cleavage, and acetal lactonization [6,25,26,27]. Here, the biosynthetic pathway of compound **1** was also proposed (Figure 7).

*Stenotrophomonas maltophilia* is an aerobic Gram-negative opportunistic pathogen and one of the most common non-fermenting Gram-negative bacilli [28]. As an ongoing effort to explore new antibiotics against *S. maltophilia*, all isolated compounds (**1**–**16**) were tested for their antibacterial activities against *S. maltophilia*. Compounds **7**–**16** showed weak antibacterial activities against *S. maltophilia* with MICs of 256, 512, 512, 256, 512, 256, 128, 512, 256, and 512 μg/mL, respectively (MICs of ceftriaxone sodium and levofloxacin were 128 and 0.25 μg/mL, respectively).

## 3. Materials and Methods

### 3.1. General Experimental Procedures

High-resolution electrospray ionization mass spectra (HR ESIMS) were recorded on a Shimadzu LC MS-IT-TOF mass spectrometer equipped with an ESI interface. Optical rotation was determined on an Autopol VI automatic polarimeter. A Chirascan-plus CD spectrometer was employed for the UV and ECD spectra detection. IR spectrum was recorded by the FT-IR-650 spectrometer as KBr disk. Nuclear magnetic resonance (NMR) spectra were measured by a Bruker AVANCE NEO 600M NMR spectrometer (^1^H: 600 MHz; ^13^C: 150 MHz). Reversed-phase semi-preparative high-performance liquid chromatography (RP-HPLC) isolation was performed on an LC 3000 system equipped with a UV detector and a Kromasil C18 column (10 mm × 250 mm, 10 μm) with flow rate of 3 mL/min. Normal phase semi-preparative HPLC (NP-HPLC) isolation was used with an AS20005 system equipped with a UV detector and silica gel column (10 mm × 250 mm, 5 μm, Hanbon Sci & Tech (Suzhou, China), with flow rate of 3 mL/min. Sephadex LH-20 was purchased from GE healthcare company. Silica gel (200–300, 300–400 mesh) for column chromatography (CC) and silica gel GF254 (10–40 μm) for thin layer chromatography (TLC) and preparative TLC were obtained from Qingdao Haiyang Chemical Co., Ltd., Qingdao, China. All solvents were of analytical grade.

### 3.2. Fungal Material

*Talaromyces amestolkiae* MST1-15 was isolated from the soil collected in the Xingren coal area of Guizhou province in China (25°21′54″ N, 105°9′9″ E), in May 2020. The soil sample was suspended in sterile water and stirred for 10 min to obtain the suspension. A serial dilution method was subsequently adopted and the aliquot from each dilution was inoculated on potato dextrose agar (PDA) plates at 28 °C for 72 h. Isolates that appeared morphologically different were selected and purified, and maintained on a PDA slant stored at 4 °C. The strain was finally identified on the basis of the morphology and ITS analysis (see Supplementary Materials S1). The fungus was deposited at the Microbiological Collection Center of Guizhou Medical University (GMU-2020-MST 1-15).

### 3.3. Fermentation, Extraction, and Isolation

The fungus was firstly cultured in potato dextrose broth in aerobic closed conical flask shaking with 140 r/min at 28 °C for 4 days. A 10% volume (vs. rice weight) of the fungi suspension was subsequently inoculated into sterile rice medium with 0.3% peptone and cultured in a 28 °C incubator for 30 days to obtain the fermentation substance.

The fermentation substance (8 kg) of *T. amestolkiae* MST1-15 was extracted with EtOAc (30 L × 2, 2 days for each time) at room temperature. The extract was evaporated under reduced pressure to obtain an ethyl acetate extract (71.0 g). The extract was subjected over a silica gel column and eluted with petroleum ether (PE)/acetone (10/1, 5/1, 3/1, 1/1, 0/1, *v*/*v*, each 2000 mL) to give five fractions (Fr. A–E) based on TLC analysis. Fr. B was further purified by Sephadex LH-20 column eluted with CHCl_3_/MeOH (3/2, *v*/*v*) to yield Fr. B1-B3. Subfraction B2 was subsequently separated by NP-HPLC eluted with PE/EtOAc (65/45, *v*/*v*) to produce compounds **1** (13 mg, t_R_ = 15 min), **2** (6 mg, t_R_ = 38 min), and **6** (0.8 mg, t_R_ = 45 min). B3 was further purified by NP-HPLC using gradient elution with PE/EtOAc (65/35→55/45, *v*/*v*) to give compounds **13** (49 mg, t_R_ = 30 min) and **14** (7 mg, t_R_ = 35 min). Fraction C was chromatographed over a silica gel column eluted with PE/EtOAc (10/1→1/1, *v*/*v*) to afford subfractions C1–C4. Compounds **3** (8 mg, t_R_ = 53 min) and **4** (0.8 mg, t_R_ = 41 min) were isolated with RP-HPLC eluting with MeOH/H_2_O (50/50, *v*/*v*) from C1. C2 was further isolated by RP-HPLC eluting with MeOH-H_2_O (45/55, *v*/*v*) to give compound **15** (13 mg, t_R_ = 36 min). C3 was subjected to RP-HPLC eluted with MeOH/H_2_O (45/55, *v*/*v*) to give compound **5** (6 mg, t_R_ = 25 min). Fraction D was purified over a silica gel column gradient elution with CH_2_Cl_2_/MeOH (40/1→5/1, *v*/*v*) to produce subfractions D1–D4. Compound **11** (3 mg) was obtained by preparative TLC developed with CH_2_Cl_2_/EtOAc (*v*/*v* = 1/1) from D1. D2 was purified by RP-HPLC eluted with MeOH/H_2_O (43/57, *v*/*v*) to give **16** (10 mg, t_R_ = 43 min). Fraction D3 was subjected to RP-HPLC using MeOH/H_2_O (47/53, *v*/*v*) as mobile phase to afford compounds **7** (7 mg, 26 min) and **18** (6 mg, 32 min). Compounds **8** (6 mg, t_R_ = 41 min), **9** (7 mg, t_R_ =50 min), and **12** (3 mg, t_R_ = 25 min) were isolated from D4 using RP-HPLC eluted with MeOH/H_2_O (40/60, *v*/*v*).

### 3.4. Spectral and Physical Data of Compounds ***1***, ***8***, and ***9***

Amestolkolide E (**1**): White amorphous powder; [α]${}_{D}^{21}$ −132.6 (c 0.11, CH_3_OH); UV (MeOH) λmax (log ε): 245 (3.67) nm; IR (KBr) υ_max_: 2962, 2923, 2805, 1671, 1619, 1459, 1382, 1234, 1112, and 790 cm^−1^; HR ESIMS (neg.) *m/z* 471.2025 [M − H]^−^ (calcd. 471.2024). CD (0.93 mM, MeOH) λ_max_ nm (Δε) 244 (−9.98).

(R)-3-((3S,4R)-3,4-dihydroxypentyl)-8-hydroxyisochroman-1-one (**8**): White amorphous powder; [α]${}_{D}^{21}$ −12.6 (c 0.23, CH_3_OH); UV (MeOH) λ_max_ (log ε): 210 (3.95), 247 (3.44), 315 (2.70) nm; IR (KBr) υ_max_: 3310, 2896, 2857, 1629, 1581, 1425, 1355, 1201, 1089, and 790 cm^−1^; HR ESIMS (neg.) *m/z* 265.1081 [M − H]^−^ (calcd. 265.1081); CD (1.11 mM, MeOH) λ_max_ nm (Δε) 216 (−5.46), 239 (+3.19), 257 (−4.94).

(R)-3-((3S,4S)-3,4-dihydroxypentyl)-8-hydroxyisochroman-1-one (**9**): White amorphous powder; [α]${}_{D}^{21}$ −26.0 (c 0.37, CH_3_OH); UV (MeOH) λ_max_ (log ε): 208 (3.98), 245 (3.40), 315 (3.22) nm; IR (KBr) υ_max_: 3232, 2911, 2865, 1633, 1585, 1428, 1303, 1201, 1085, 960, and 786 cm^−1^; HR ESIMS (neg.) *m/z* 265.1084 [M − H]^−^ (calcd. 265.1081); CD (0.98 mM, MeOH) λ_max_ nm (Δε) 217 (−1.56), 239 (+0.88), 258 (−1.48).

### 3.5. Antibacterial Assay

The minimum inhibitory concentrations (MICs) were evaluated according to the reported procedure [29,30] with minor modifications. Briefly, *Stenotrophomonas maltophilia* ATCC 13637 was cultured on 0002 medium (1% peptone, 0.3% beef extract, and 0.5% NaCl in distilled water with pH 7.0) at 30 °C and prepared for its suspension with a final concentration of 5 × 10^5^ CFU/mL. Samples (**1**–**16**) were severally added into 96-well plates employing serial two-fold dilution from concentrations of 512 to 2 μg/mL. Whereafter, 100 μL bacterial suspension was transferred to the wells to incubate for 16 h at 30 °C. The final concentration of 0.25% TTC (2,3,5-triphenyl tetrazolium chloride, Sigma) in wells as microorganism growth indicator was added and the microorganism was continuously incubated for 3 h, and the concentration in well that showed no red color indicating complete growth inhibition of bacteria was determined as MIC. Equivalent amounts of DMSO (2.5%) and medium were successively used as negative and blank controls. Ceftriaxone sodium and levofloxacin were used as positive controls. Each experiment was performed in triplicate.

### 3.6. Preparation of Acetonides of ***8a*** and ***9a***

To a solution of compounds **8** and **9** (3.0 mg) in 200 μL DMF was added 2,2-dimethoxypropane (2 equiv) and *p*-TsOH (0.5 equiv), respectively [18], and the mixtures were stirred at room temperature for 6 h. The reaction was quenched by saturated aqueous NaHCO_3_ (200 μL) and extracted with EtOAc (500 μL × 3). The organic layer was dried with Na_2_SO_4_ and evaporated under reduced pressure. The residue was subjected to silica gel column chromatography eluted with petroleum ether/ethyl acetate (4:1) to afford **8a** (2.6 mg) and **9a** (2.9 mg).

Compound **8a**: ^1^H NMR (600 MHz, CD_3_OD) δ 7.46 (1H, dd, *J* = 8.0, 7.4 Hz, H-6), 6.85 (1H, d, *J* = 8.0 Hz, H-7), 6.80 (1H, d, *J* = 7.4 Hz, H-5), 4.69 (1H, m, H-3), 4.30 (1H, m, H-4′), 4.12 (1H, m, H-3′), 3.05 (1H, dd, *J* = 16.4, 3.4 Hz, H-4), 2.98 (1H, dd, *J* = 16.4, 11.2 Hz, H-4), 2.05 (1H, m, H-1′), 1.81 (1H, m, H-1′), 1.74 (1H, m, H-2′), 1.62(1H, m, H-2′), 1.42 (3H, s, CH_3_-8′), 1.32 (3H, s, CH_3_-7′), 1.18 (3H, d, *J* = 6.4 Hz, H-5′). ^13^C NMR (150 MHz, CD_3_OD) δ 171.4 (C-1), 163.2 (C-8), 141.6 (C-10), 137.4 (C-6), 119.4 (C-5), 116.7 (C-7), 109.5 (C-9), 108.7 (C-6′), 80.9 (C-3), 78.8 (C-3′), 75.1 (C-4′), 33.7 (C-4), 32.6 (C-1′), 28.8 (CH_3_-8′), 26.3 (C-2′), 26.0 (CH_3_-7′), 15.7 (C-5′). ESI MS (neg.) *m/z* 305.1 [M − H]^−^.

Compound **9a**: ^1^H NMR (600 MHz, CD_3_OD) δ 7.43 (1H, dd, *J* = 8.0, 7.4 Hz, H-6), 6.82 (1H, d, *J* = 8.0 Hz, H-7), 6.77 (1H, d, *J* = 7.4 Hz, H-5), 4.62 (1H, m, H-3), 3.73 (1H, m, H-4′), 3.55 (1H, m, H-3′), 3.02 (1H, dd, *J* = 16.4, 3.5 Hz), 2.95 (1H, dd, *J* = 16.4, 11.4 Hz, H-4), 1.95 (1H, m, H-1′), 1.87 (2H, m, H-1′, 2′), 1.60 (1H, m, H-2′), 1.33 (3H, s, CH_3_-7′), 1.32 (3H, s, CH_3_-8′), 1.23 (3H, d, *J* = 6.0 Hz, H-5′). ^13^C NMR (150 MHz, CD_3_OD) δ 171.4 (C-1), 163.2 (C-8), 141.6 (C-10), 137.4 (C-6), 119.4 (C-5), 116.8 (C-7), 109.5 (C-9), 109.1 (C-6′), 83.6 (C-3′), 81.4 (C-3), 78.2 (C-4′), 33.7 (C-4), 32.8 (C-1′), 28.9 (C-2′), 27.6 (CH_3_-7′), 27.5 (CH_3_-8′), 17.7 (C-5′). ESI MS (neg.) *m/z* 305.0 [M − H]^−^.

## 4. Conclusions

A chemical study of *Talaromyces amestolkiae* MST1-15 collected from coal areas led to the isolation and identification of seven meroterpenoids (**1**–**7**) and eleven isocoumarins (**8**–**16**), including three new compounds named amestolkolide E (**1**), (*R*)-3-((3*S*,4*R*)-3,4-dihydroxypentyl)-8-hydroxyisochroman-1-one (**8**), and (*R*)-3-((3*S*,4*S*)-3,4-dihydroxypentyl)-8-hydroxyisochroman-1-one (**9**). Compounds **7**–**16** showed weak antibacterial activities against *S. maltophilia* with MIC values ranging from 256 to 512 μg/mL. This study enriched the chemical structure and biological activity from the secondary metabolites of *T. amestolkiae*.

## Figures and Tables

**Table 1:** ^1^H (600 MHz) and ^13^C NMR (150 MHz) data of **1**.

No.	1 (in CDCl_3_) *^a^*	
	δ_H_ (*J* in Hz)	δ_C_
1		169.4
2	3.32 (1H, d, 21.1) 2.88 (1H, dd, 21.1, 1.3)	31.6
3		126.9
4		132.8
5		137.0
6	2.74 (1H, d, 16.2) 2.14 (1H, d, 16.2)	34.0
7		41.0
8		179.8
9	4.18 (1H, q, 6.8)	72.3
10		209.4
11		63.2
12		44.1
13	3.22 (1H, dt, 13.7, 3.6) 1.54 (1H, dd, 13.7, 3.2)	49.1
14	2.70 (1H, t like) 1.95 (1H, dt, 13.7, 3.2)	26.7
15		136.3
16		85.6
17	1.57 (3H, s)	27.4
18	1.48 (3H, s)	27.9
19	1.14 (3H, s)	27.3
20		168.2
21	1.42 (3H, d, 6.8)	17.7
22	3.69 (1H, d, 8.2)	46.0
23	6.09 (1H, d, 8.2)	98.5
24	1.33 (3H, s)	26.5
25	1.77 (3H, s)	18.3
26	3.80 (3H, s)	53.2

*^a^* All assignments are based on HSQC and HMBC experiments.

**Table 2:** ^1^H (600 MHz) and ^13^C NMR (150 MHz) data of compounds **8** and **9** (in methanol-*d_4_*) *^a^*.

No.	*δ*_H_ (*J* in Hz)		δ_C_	
	8	9	8	9
1			171.5	171.5
3	4.63 (1H, m)	4.64 (1H, m)	81.2	81.7
4	3.01 (1H, dd, 16.2, 3.0) 2.94 (1H, dd, 16.2, 11.2)	3.05 (1H, dd, 16.3, 3.2) 2.96 (1H, dd, 16.3, 11.4)	33.6	33.7
4a			141.6	141.6
5	6.76 (1H, d, 7.3)	6.80 (1H, d, 7.4)	119.3	119.3
6	7.42 (1H, dd, 8.3, 7.3)	7.45 (1H, dd, 8.4, 7.4)	137.4	137.4
7	6.81 (1H, d, 8.3)	6.84 (1H, d, 8.4)	116.7	116.7
8			163.2	163.2
8a			109.5	109.5
1′	2.05 (1H, m) 1.77 (1H, m)	2.00 (1H, m) 1.89 (1H, m)	32.3	32.6
2′	1.77 (1H, m) 1.57 (1H, m)	1.82 (1H, m) 1.53 (1H, m)	28.9	29.0
3′	3.35 (1H, m)	3.38 (1H, m)	76.2	76.3
4′	3.55 (1H, m)	3.63 (1H, m)	71.8	71.4
5′	1.16 (3H, d, 6.3)	1.17 (3H, d, 6.4)	18.8	18.9

*^a^* All assignments are based on HSQC and HMBC experiments.

## Data Availability

Not applicable.

## Supplementary Materials

The following supporting information can be downloaded at: https://www.mdpi.com/article/10.3390/molecules27238223/s1. References [31,32] are cited in the Supplementary Materials.

## Sample Availability

Samples of the compounds are not available from the authors.
