# Supplementary material for: New Meroterpenoid and Isocoumarins from the Fungus Talaromyces amestolkiae MST1-15 Collected from Coal Area
Source: Molecules. 2022 Nov 25;27(23):8223. doi: 10.3390/molecules27238223 (PMC9741378; doi:10.3390/molecules27238223)
Supplement: Supplementary file 1 [file molecules-27-08223-s001.zip › molecules-1973330-supplementary.pdf]

molecules

MDPI

Supplementary Materials

New Meroterpenoid and Isocoumarins from the Fungus *Talaromyces amestolkiae* MST1-15 Collected from Coal Area

Kai-Yu Li [1,†], Qin-Feng Zhu [1,†], Jun-Li Ao [1,2], Fu-Rui Wang [1], Xing-Mei Long [1], Shang-Gao Liao [1,2,*] and Guo-Bo Xu [1,2,*]

1 State Key Laboratory of Functions and Applications of Medicinal Plants, School of Pharmacy, Guizhou Medical University, Guian New District, Guiyang 550025, China
2 Engineering Research Center for the Development and Application of Ethnic Medicine and TCM, Ministry of Education & Guizhou Provincial Key Laboratory of Pharmaceutics, Guiyang 550004, China
* Correspondence: lshangg@163.com (S.-G.L.); xguobo@163.com (G.-B.X.)
† These authors contributed equally to this work.

Contents of Supporting Information

S1. Fungal material identification 4
Figure S1. Structure of compounds **1–16** 6
Figure S2. HR ESIMS of **1** 6
Figure S3. UV spectrum of **1** 7
Figure S4. IR spectrum of **1** 7
Figure S5. ^{1}H NMR spectrum of **1** 8
Figure S6. ^{13}C NMR spectrum of **1** 8
Figure S7. HSQC spectrum of **1** 9
Figure S8. HMBC spectrum of **1** 9
Figure S9. ^{1}H-^{1}H COSY spectrum of **1** 10
Figure S10. NOESY spectrum of **1** 10
Figure S11. HR ESIMS of **8** 11
Figure S12. UV spectrum of **8** 11
Figure S13. IR spectrum of **8** 12
Figure S14. ^{1}H NMR spectrum of **8** 12
Figure S15. ^{13}C NMR spectrum of **8** 13
Figure S16. HSQC spectrum of **8** 13
Figure S17. HMBC spectrum of **8** 14
Figure S18. ^{1}H-^{1}H COSY spectrum of **8** 14
Figure S19. ESI-MS of **8a** 15
Figure S20. ^{1}H NMR spectrum of **8a** 15
Figure S21. ^{13}C NMR spectrum of **8a** 16
Figure S22. NOESY spectrum of **8a** 16
Figure S23. HR ESIMS of **9** 17
Figure S24. UV spectrum of **9** 17
Figure S25. IR spectrum of **9** 18
Figure S26. ^{1}H NMR spectrum of **9** 18
Figure S27. ^{13}C NMR spectrum of **9** 19
Figure S28. HSQC spectrum of **9** 19
Figure S29. HMBC spectrum of **9** 20
Figure S30. ^{1}H-^{1}H COSY spectrum of **9** 20
Figure S31. ESI-MS of **9a** 21
Figure S32. ^{1}H NMR spectrum of **9a** 21
Figure S33. ^{13}C NMR spectrum of **9a** 22
Figure S34. NOESY spectrum of **9a** 22
Figure S35. Isomers (at C_9 and C_{11}) of compound **1** 23
Table S1. Parameters of the calculated chemical shifts (C data) of **1a-1d** 23

S1. Fungal material identification

Talaromyces amestolkiae MST1-15 was identified by morphology and ITS methods.

Morphology analysis (Figure 1a) suggested strain of MST1-15 belong to the genus *Talaromyces* comparing that report [1]. ITS analysis [2]: Fungal hyphae were suspended in 50 μl of a lysis solution containing 100 mmol l^{-1} Tris-HCl, pH 8.0, 10 mmol l^{-1} EDTA. Then heated in a microwave oven at 600～700 W for 45 s and transferred to ice bath for 2 minutes immediately, centrifuged at 12,000 rpm for 2 min at room temperature, and the supernatant was used as template to amplify the non-coding regions of the conserved regions by using the universal ITS primers ITS1 (5′-TCC GTA GGT GAA CCT GCG G-3′) and ITS4 (5′-TCC TCC GCT TAT TGA TAT GC-3′). The PCR amplification conditions consisted of 3 min at 95°C, followed by 35 cycles of 1 min at 92°C, 1 min at 50°C, and 2 min at 72°C; and with a final extension of 10 min at 72°C in an Eppendorf thermal cycler (Mastercycler pro 384, Hamburg, Germany). The PCR products were analyzed by agarose gel electrophoresis (Figure 2a) and purified by using a universal DNA purification kit (Tiangen Biotech Co., Ltd., Beijing, China). The purified products were sequenced on a 3730XL DNA sequencer (Applied Biosystem, USA). The resulting consensus sequence is as follows:

GACCTGCGGAAGGATCATTACCGAGTGCGGGCCCTCGTGGCCCAACCTCC
CACCCTTGTCTCTATACACCTGTTGCTTTGGCGGGCCCACCGGGGCCACCTGGT
CGCCGGGGGACATCTGTCCCCGGGCCCGCGCCCGCCGAAGCGCTCTGTGAACC
CTGATGAAGATGGGCTGTCTGAGTACTATGAAAATTGTCAAAACTTTCAACAA
TGGATCTCTTGGTTCCGGCATCGATGAAGAACGCAGCGAAATGCGATAAGTAA
TGTGAATTGCAGAATTCCGTGAATCATCGAATCTTTGAACGCACATTGCGCCCC
CTGGCATTCCGGGGGGCATGCCTGTCCGAGCGTCATTTCTGCCCTCAAGCACGG
CTTGTGTGTTGGGTGCGGTCCCCCCGGGGACCTGCCCGAAAGGCAGCGGCGAC-
GTCCGTCTGGTCCTCGAGCGTATGGGGCTTTGTCACTCGCTCGGGAAGGACTGGC
GGGGGTTGGTCACCACCAAAATTTTACCACGGTTGACCTCGGATCAGGTAGGAG-
TTACCCGCTGAACTTAAGCATATCAATAAGGCGGAGGA

The strain of MST1-15 was finally identified as *Talaromyces amestolkiae* by comparing the sequence with deposited sequences using BLAST and phylogenetic tree (Figure 3a)

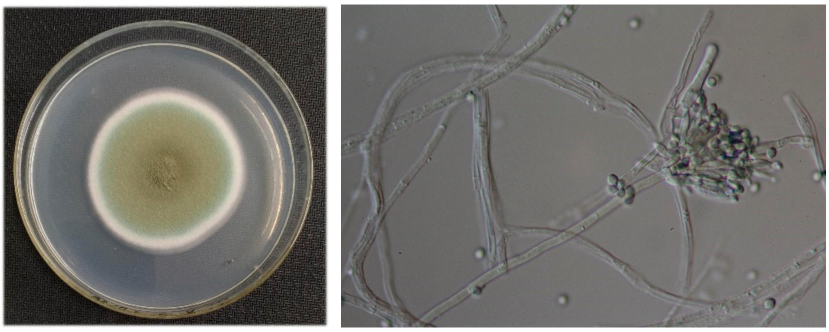

Scheme S1. The morphological characters of *Talaromyces amestolkiae* MST1-15.

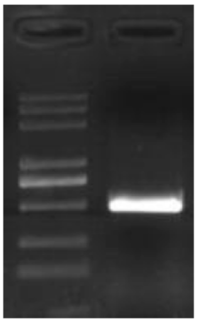

Scheme S2. ITS gel electrophoresis image.

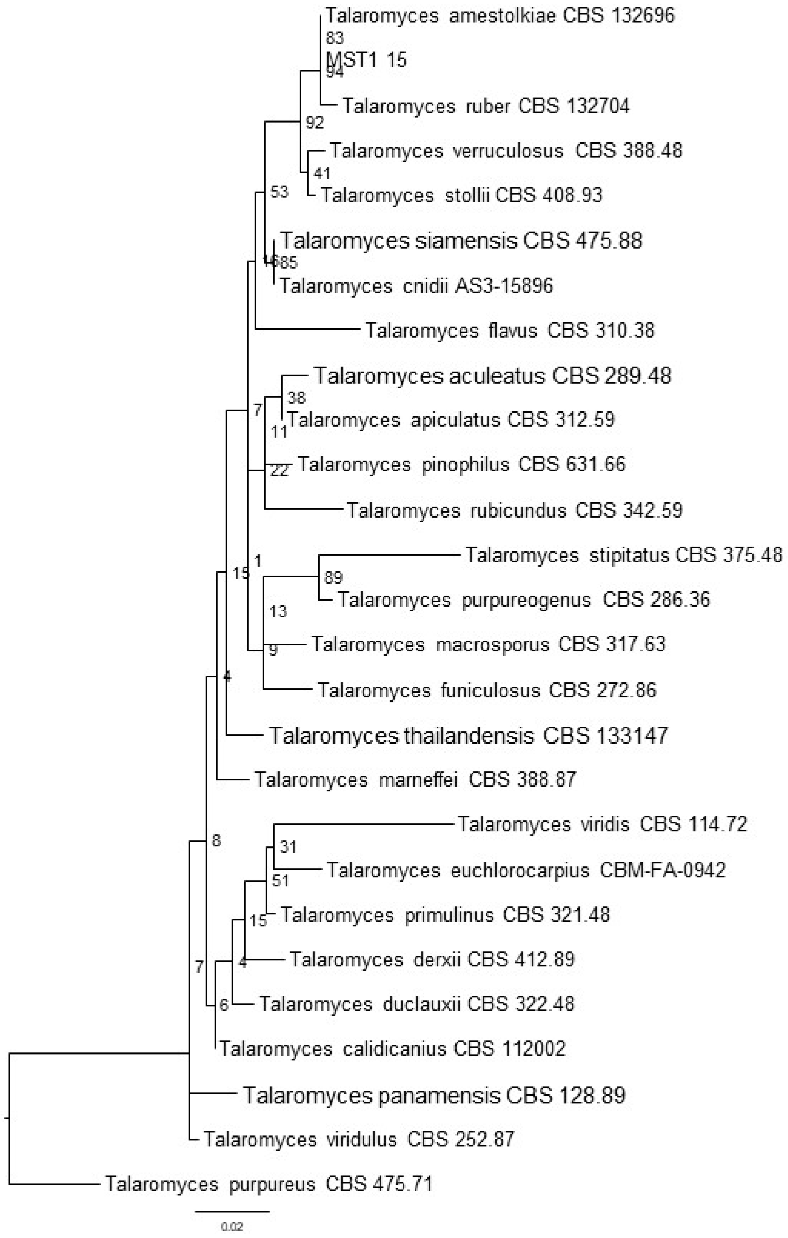

Scheme S3. Phylogenetic tree of strain identification based on ITS sequence.

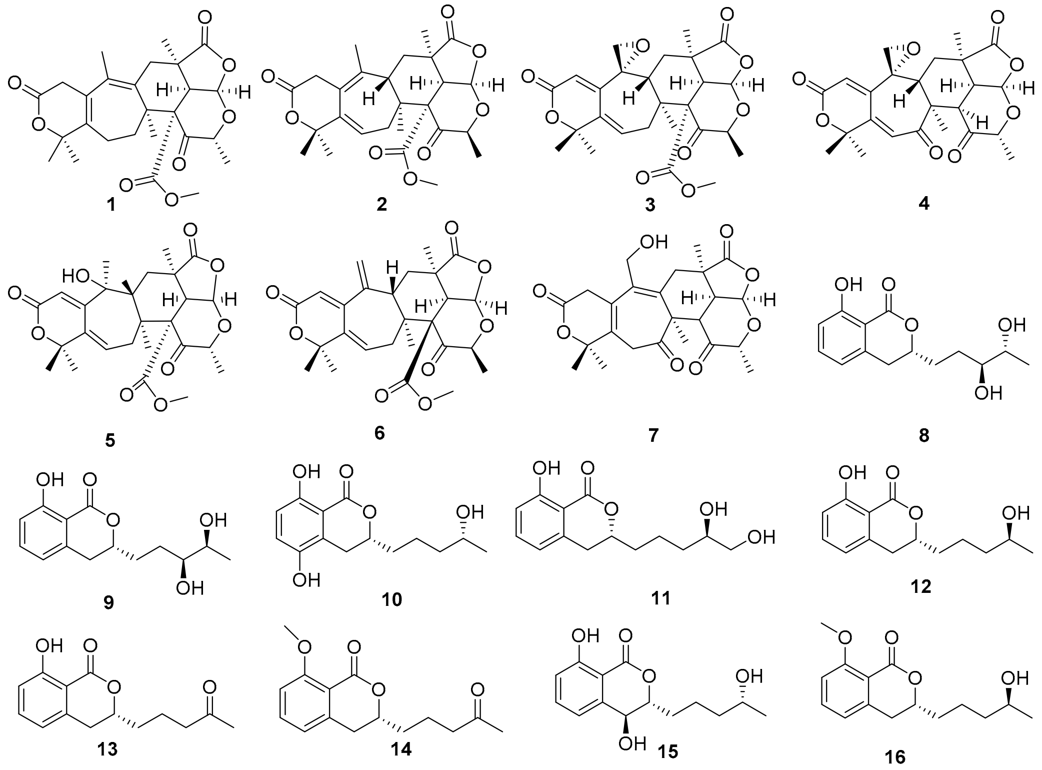

Figure S1. Structure of compounds **1–16.**

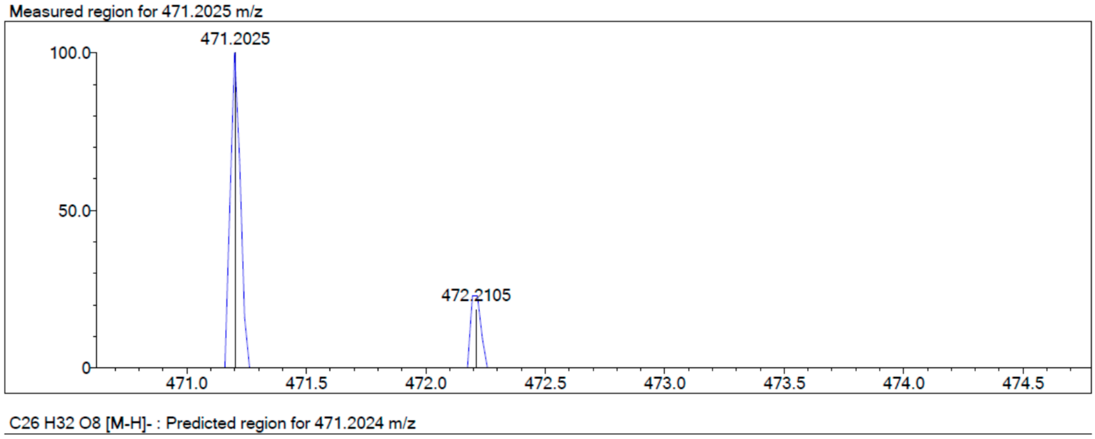

Figure S2. HR ESIMS of **1.**

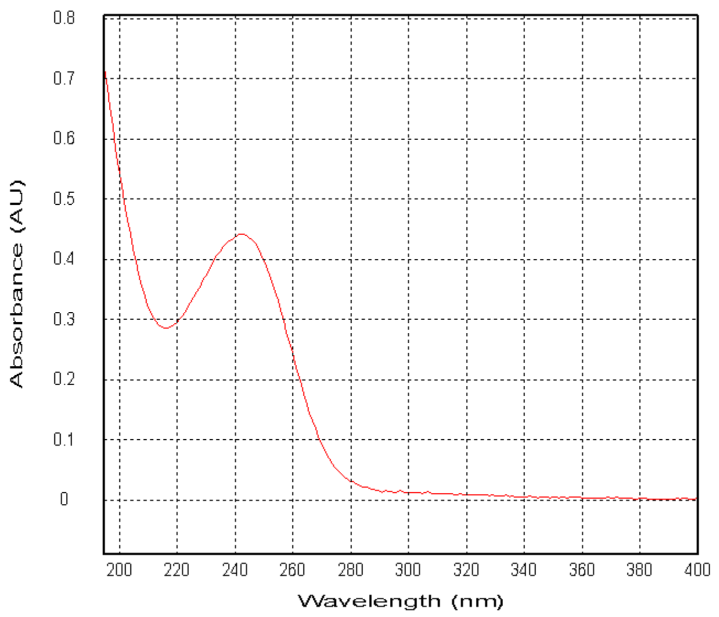

Figure S3. UV spectrum of **1.**

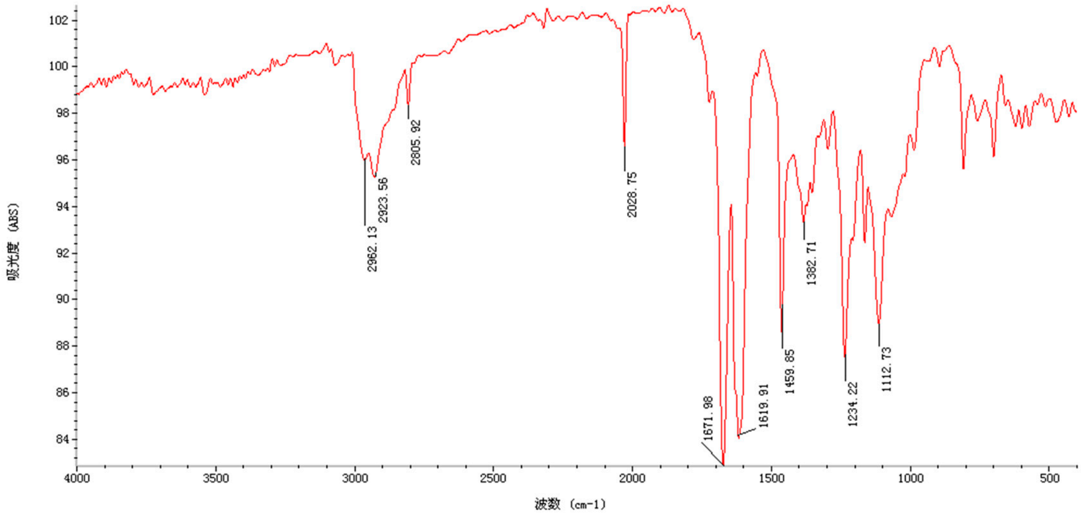

Figure S4. IR spectrum of **1.**

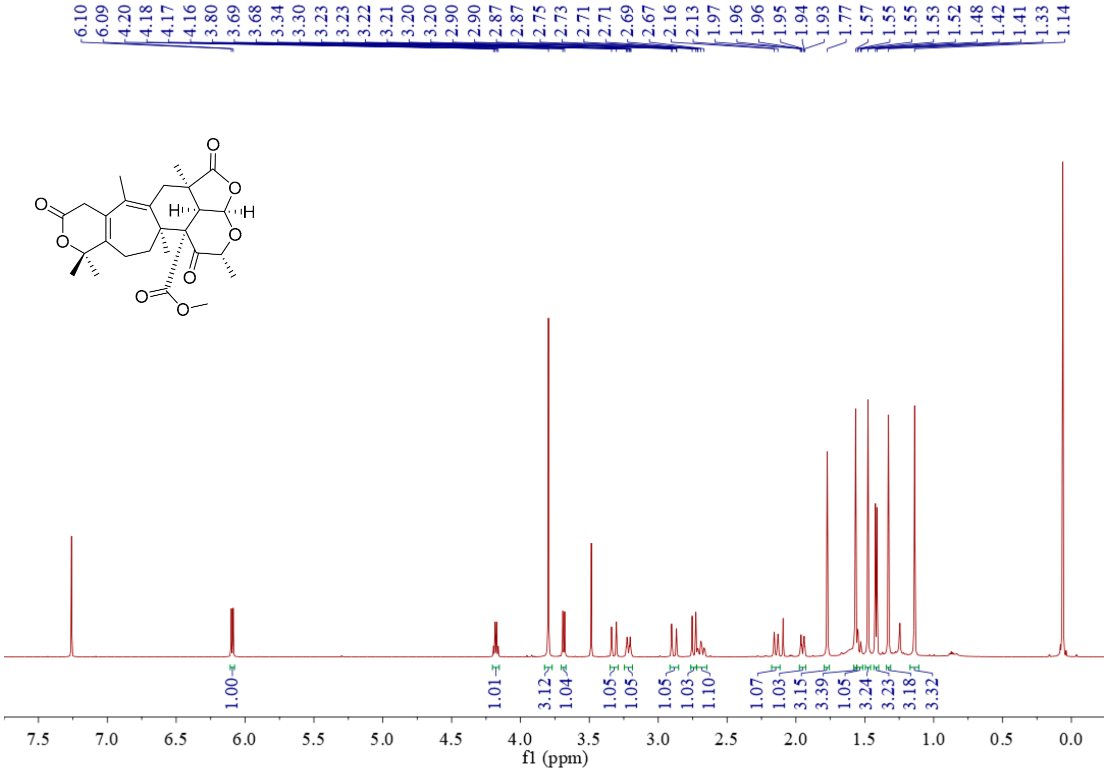

Figure S5. ^{1}H NMR spectrum of **1** (600 MHz, $CDCl_3$).

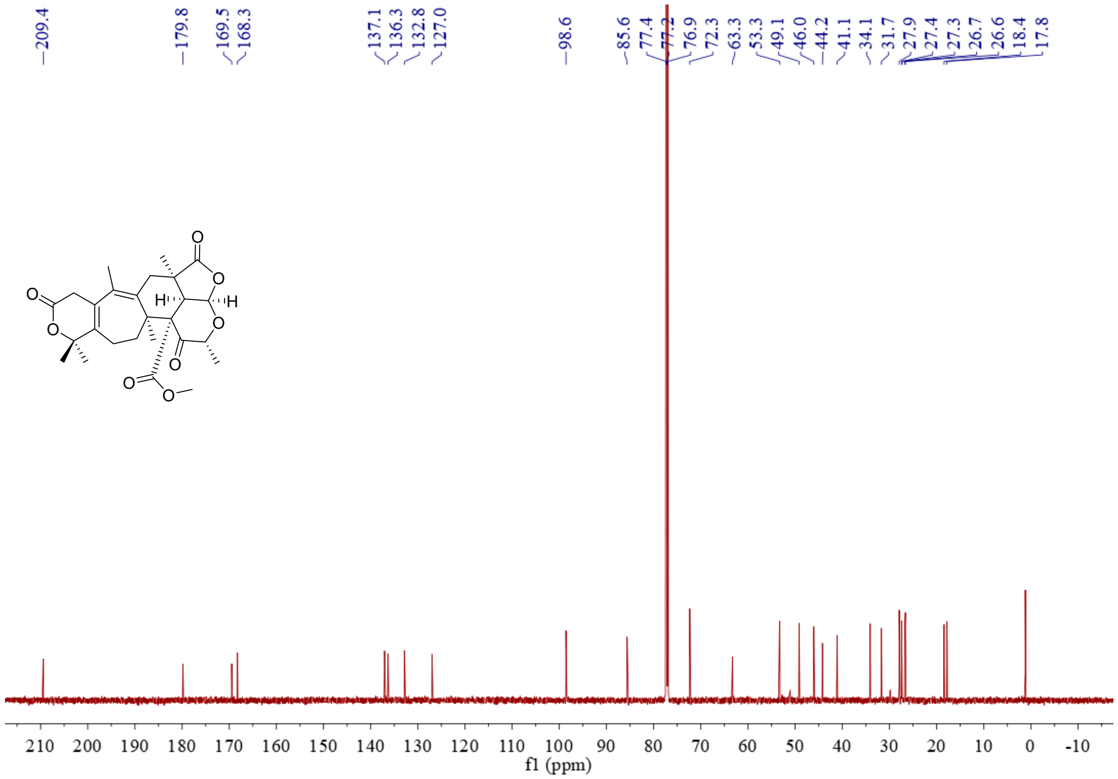

Figure S6. ^{13}C NMR spectrum of **1** (150 MHz, $CDCl_3$).

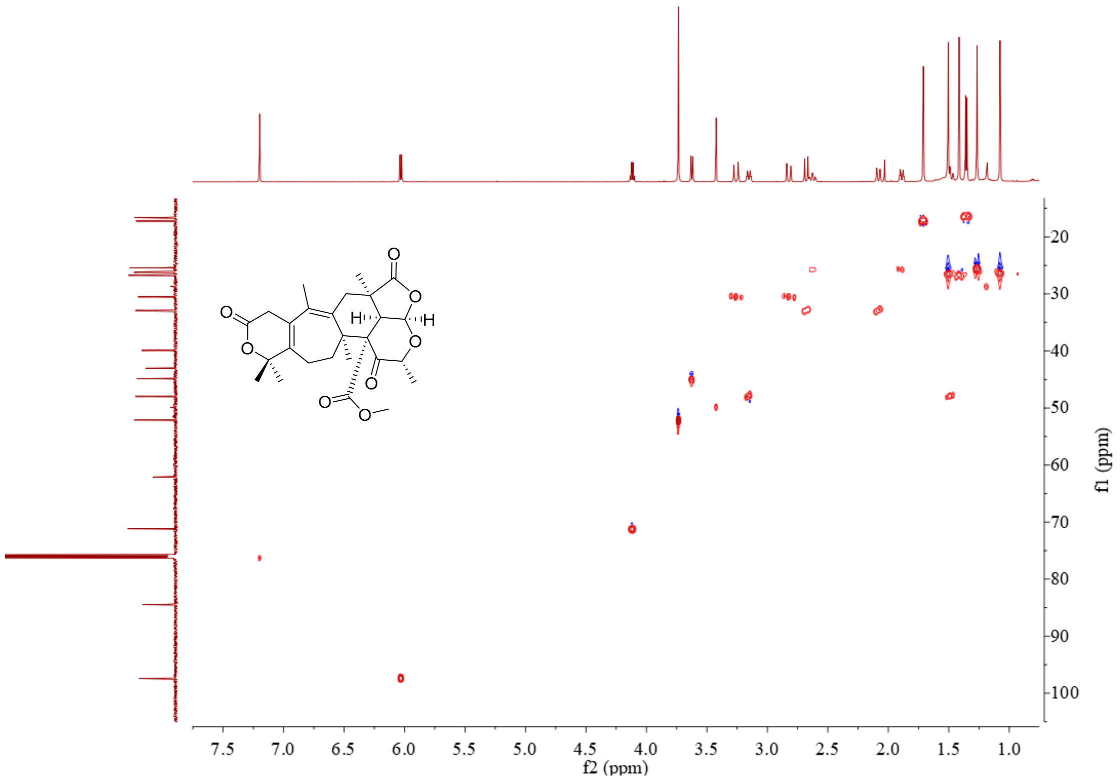

Figure S7. HSQC spectrum of **1** (600 MHz, $CDCl_3$).

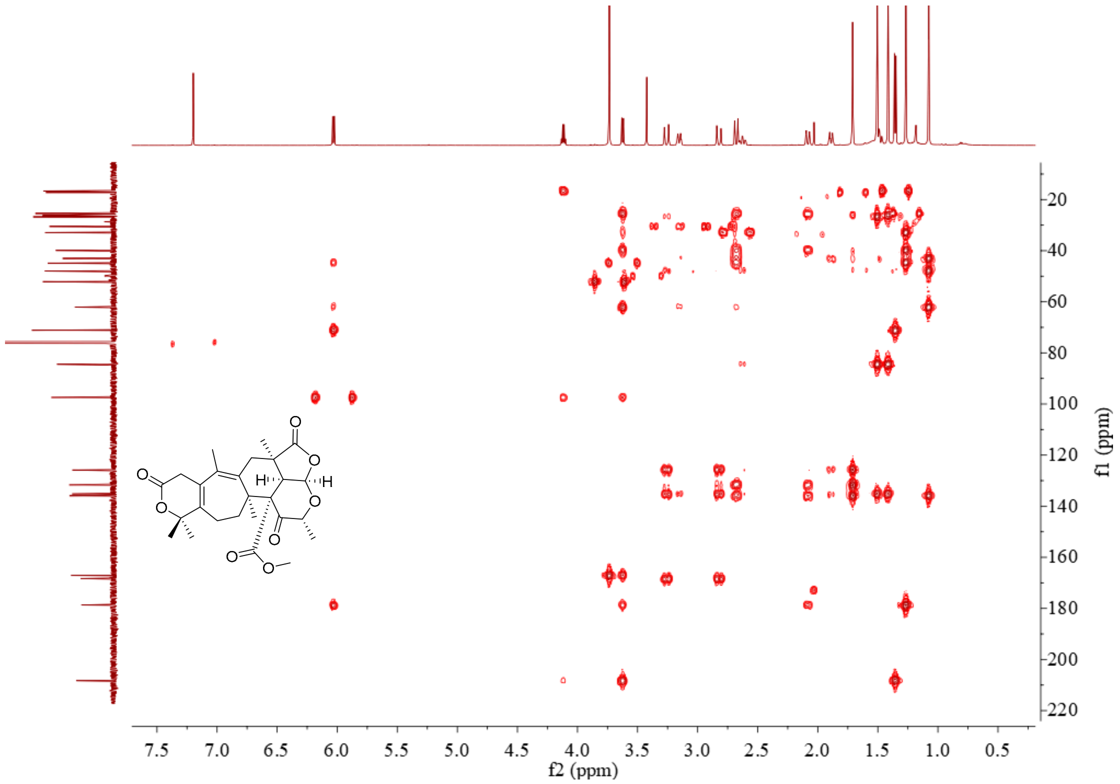

Figure S8. HMBC spectrum of **1** (600 MHz, $CDCl_3$).

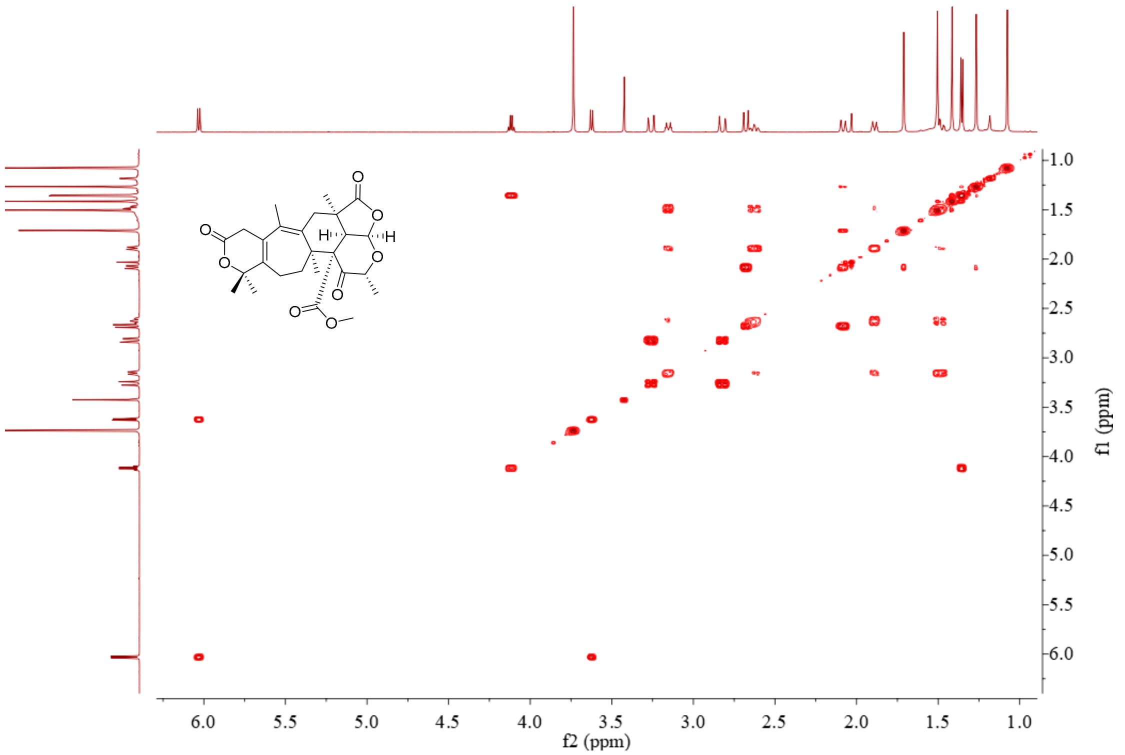

Figure S9. ^{1}H-^{1}H COSY spectrum of **1** (600 MHz, $CDCl_3$).

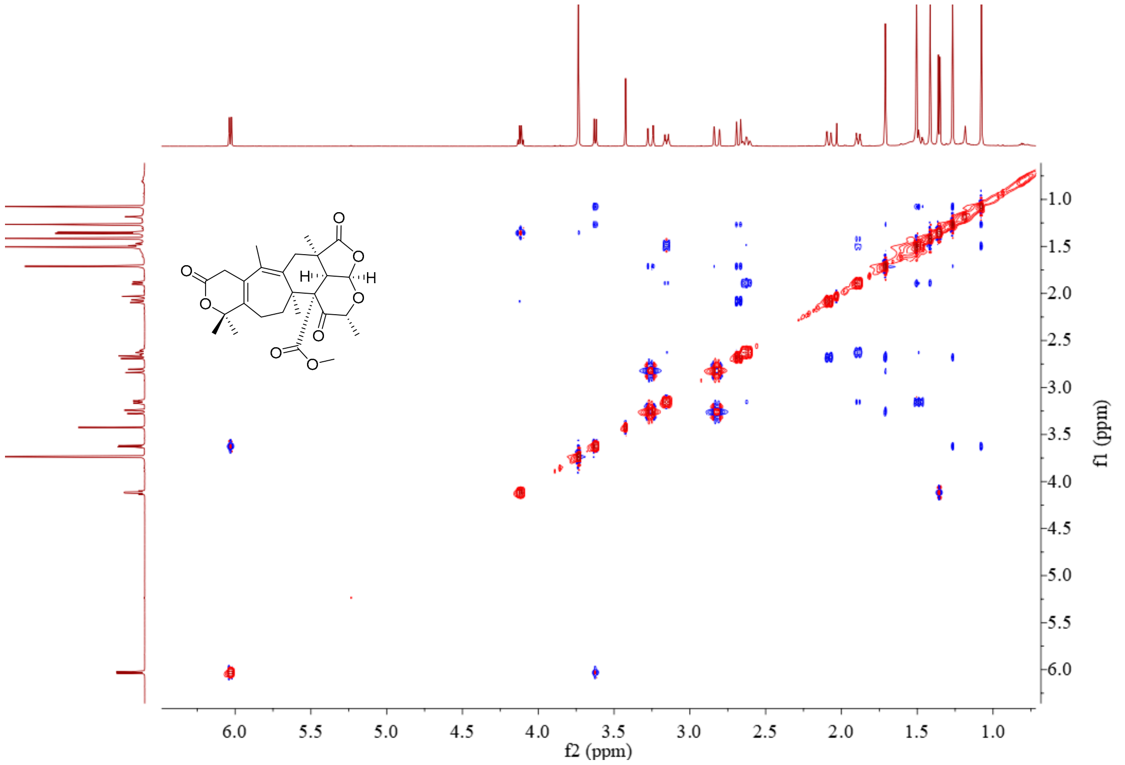

Figure S10. NOESY spectrum of **1** (600 MHz, $CDCl_3$).

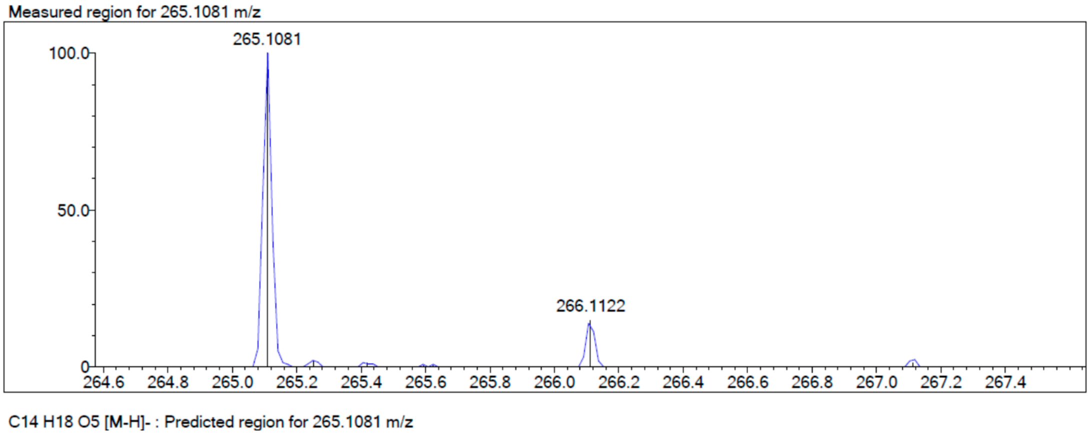

Figure S11. HR ESIMS of **8.**

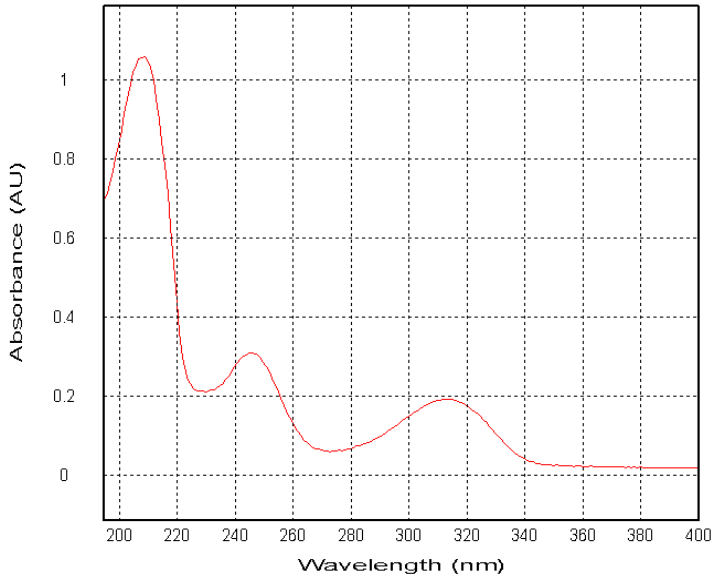

Figure S12. UV spectrum of **8.**

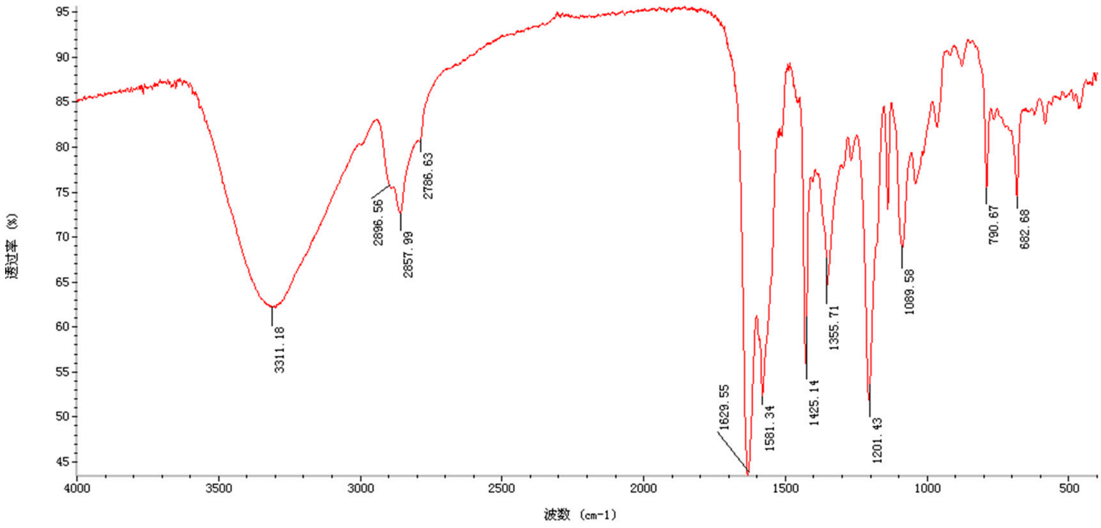

Figure S13. IR spectrum of **8.**

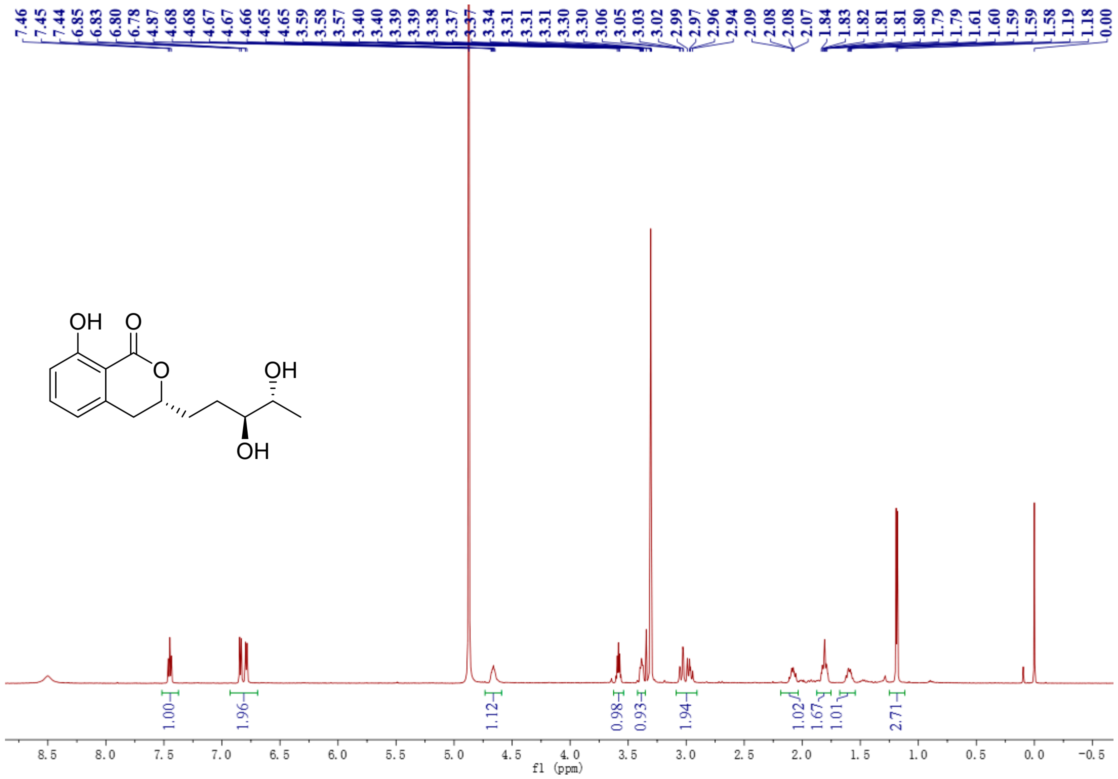

Figure S14. ^1H NMR spectrum of **8** (600 MHz, CD_3OD).

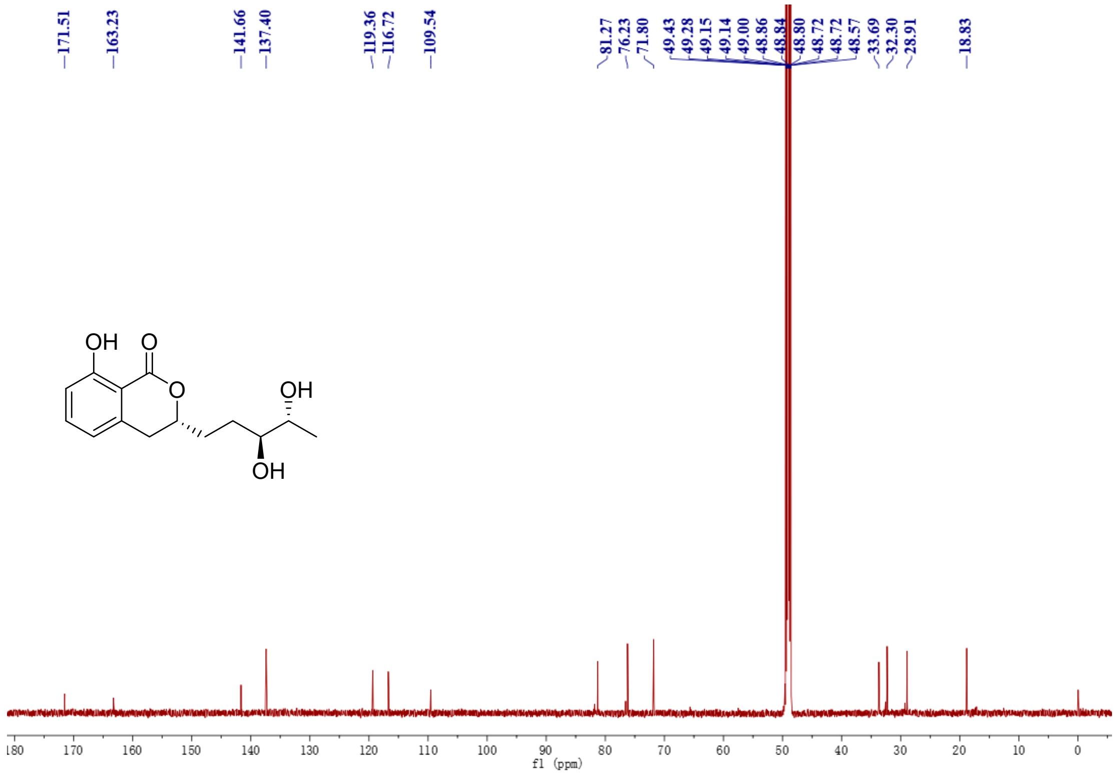

Figure S15. ^{13}C NMR spectrum of **8** (150 MHz, CD_3OD).

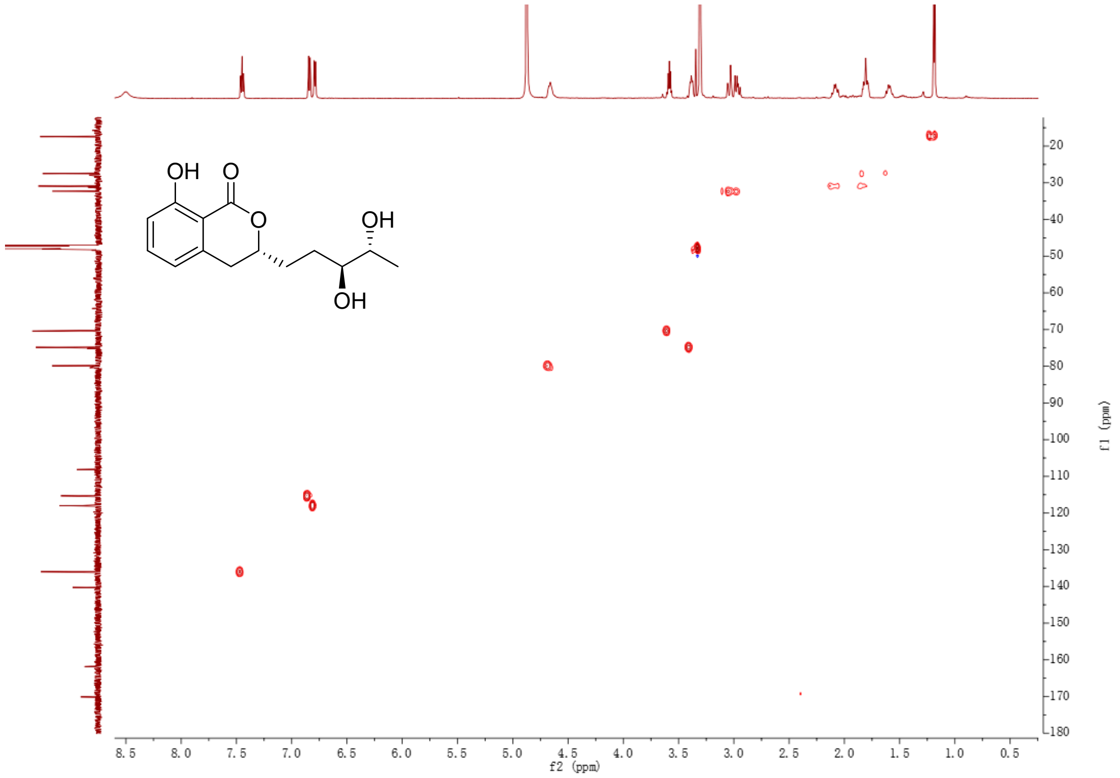

Figure S16. HSQC spectrum of 8 (600 MHz, CD3OD).

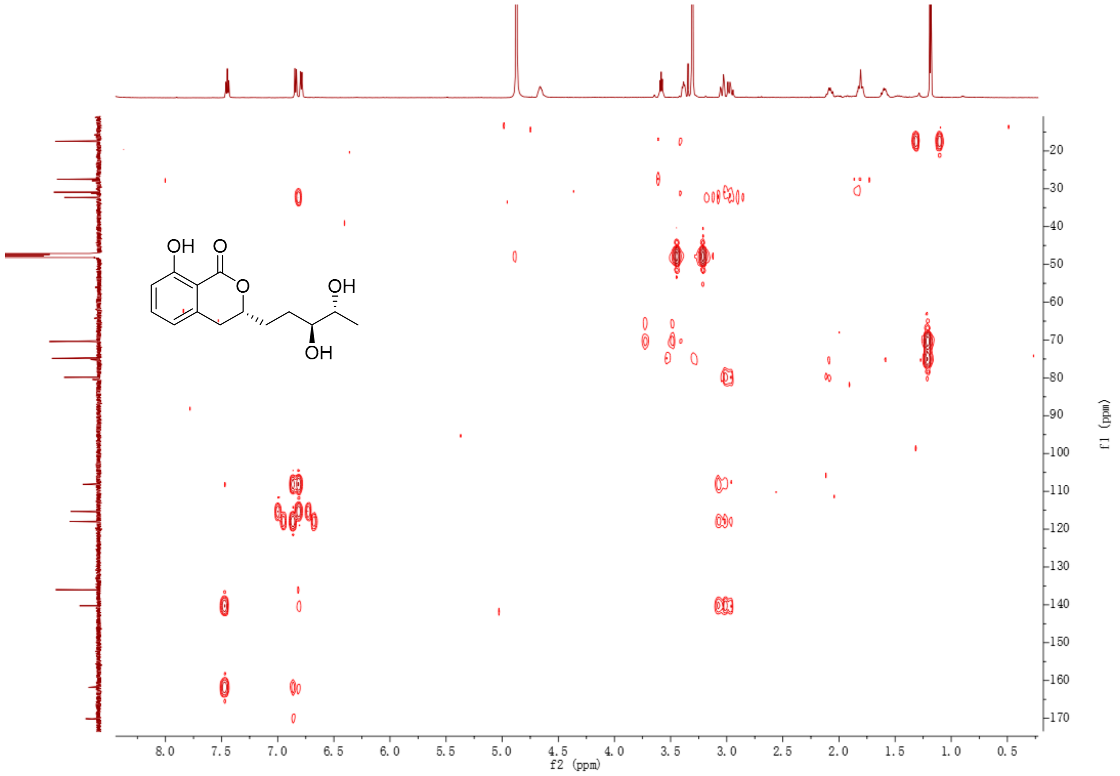

Figure S17. HMBC spectrum of **8** (600 MHz, CD3OD).

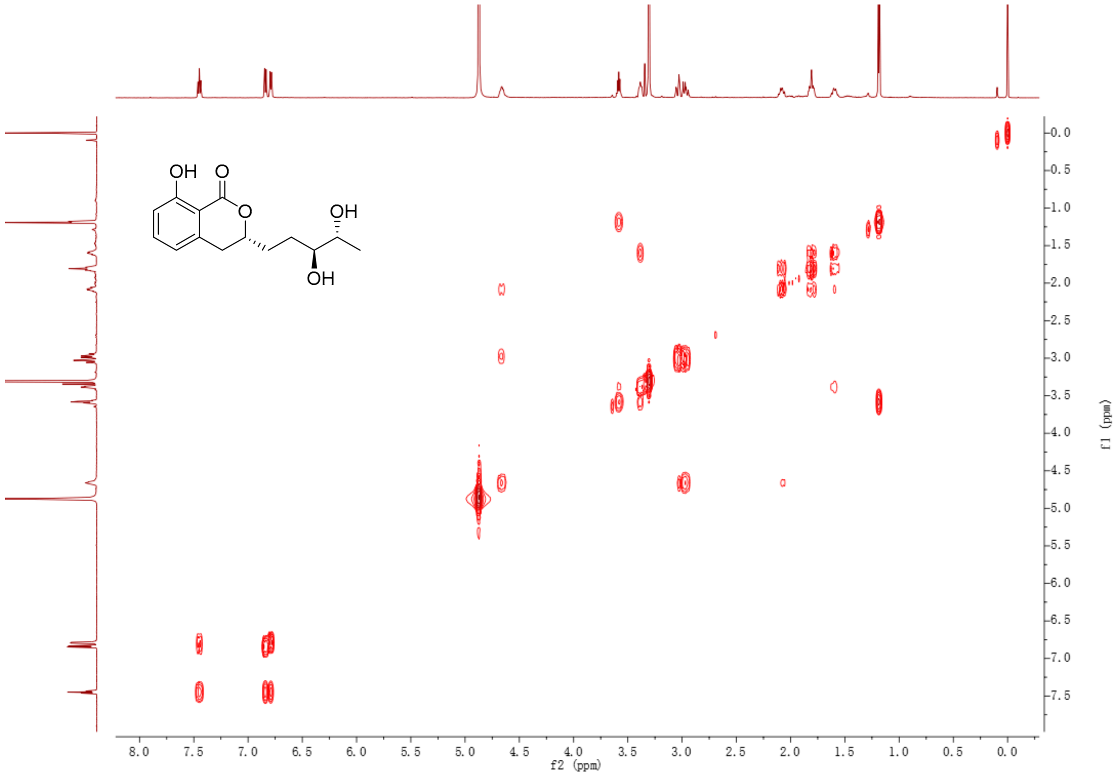

Figure S18. ^{1}H-^{1}H COSY spectrum of **8** (600 MHz, CD_3OD).

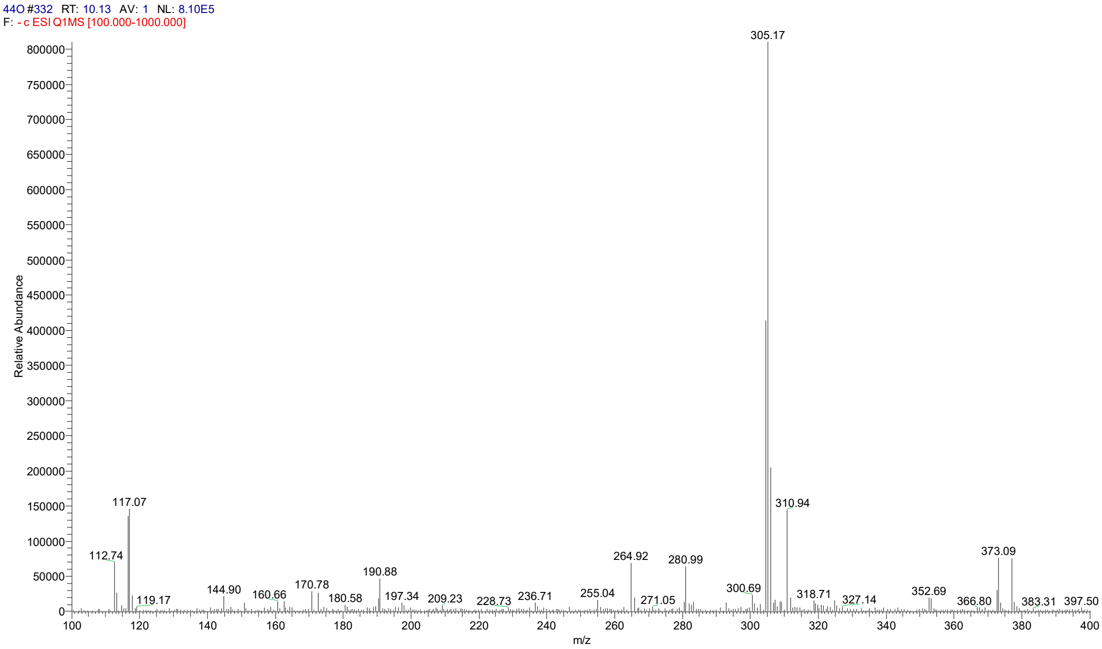

Figure S19. ESI-MS of **8a** (neg.).

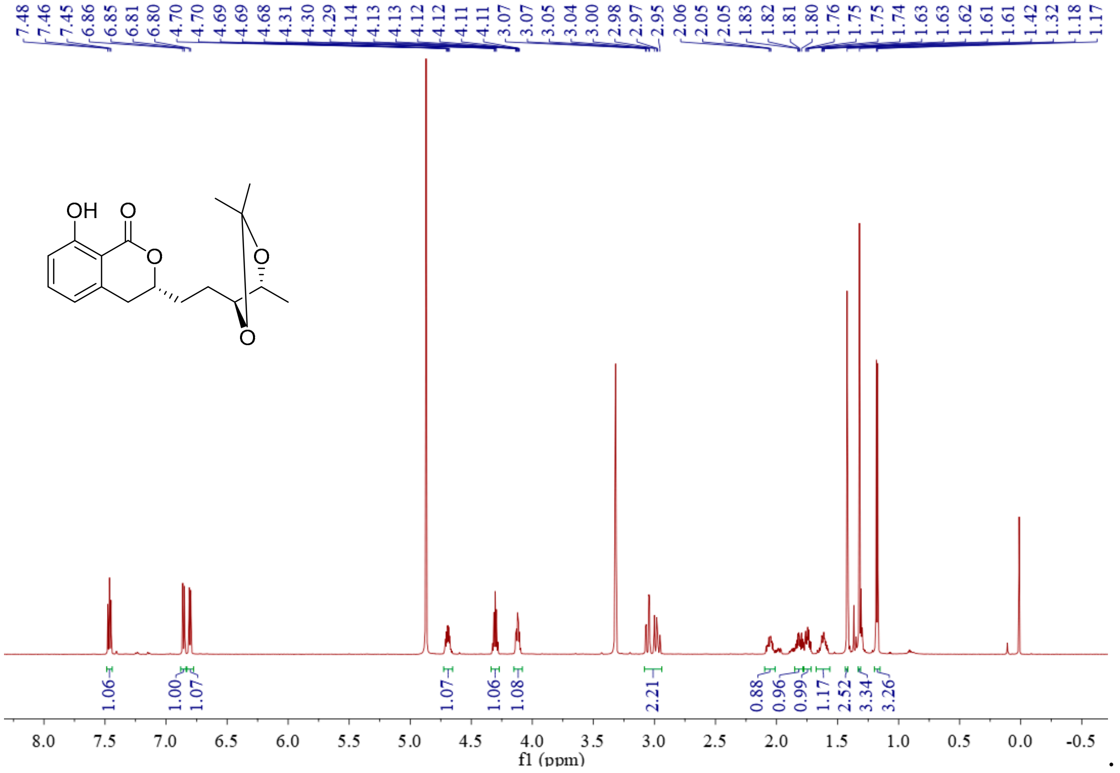

Figure S20. ^{1}H NMR spectrum of **8a** (600 MHz, CD_3OD).

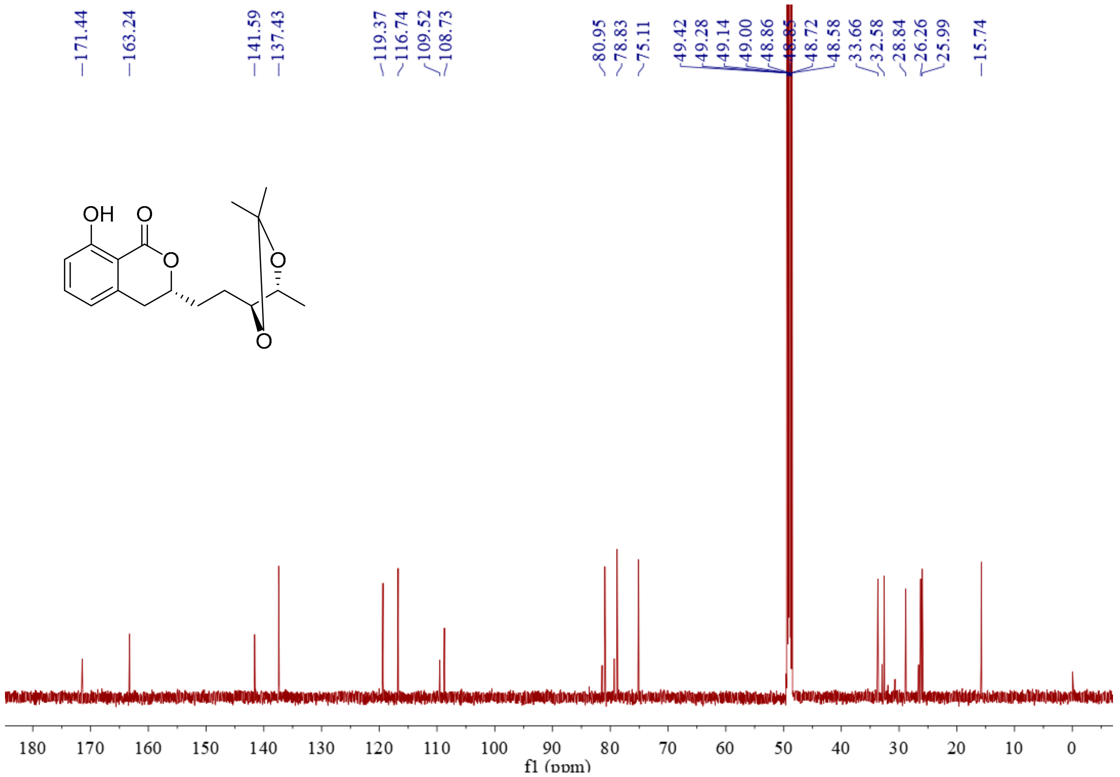

Figure S21. ^{13}C NMR spectrum of **8a** (150 MHz, CD_3OD).

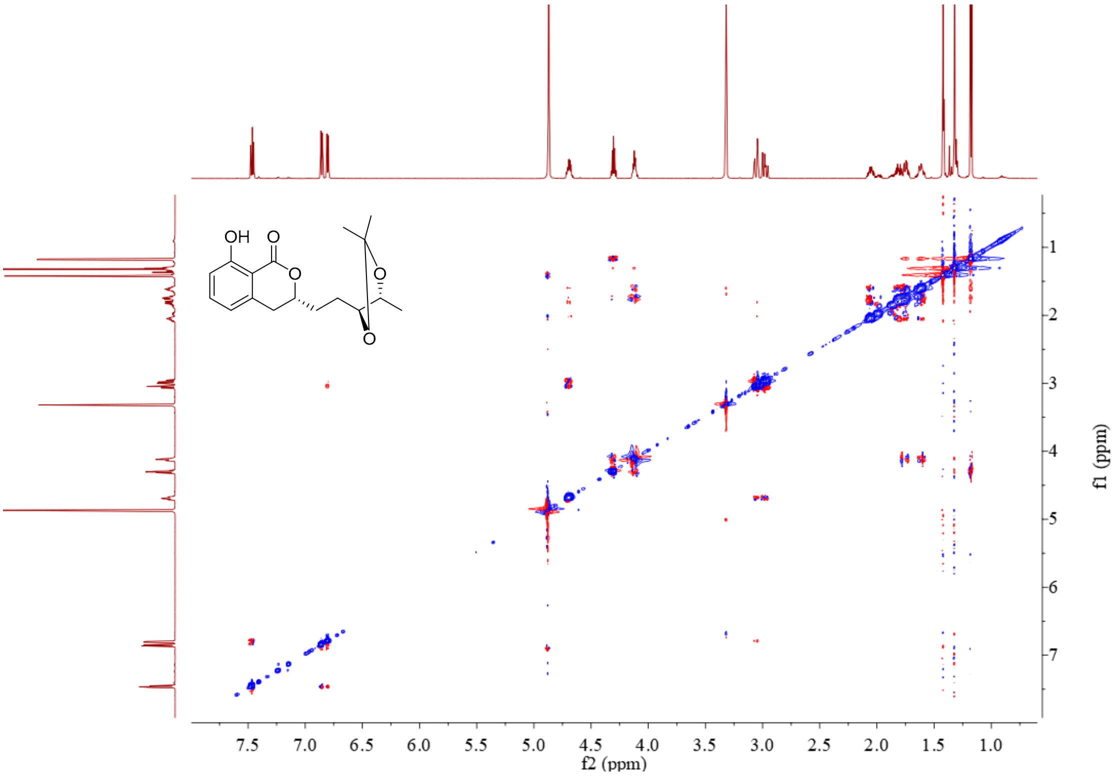

Figure S22. NOESY spectrum of **8a** (600 MHz, CD3OD).

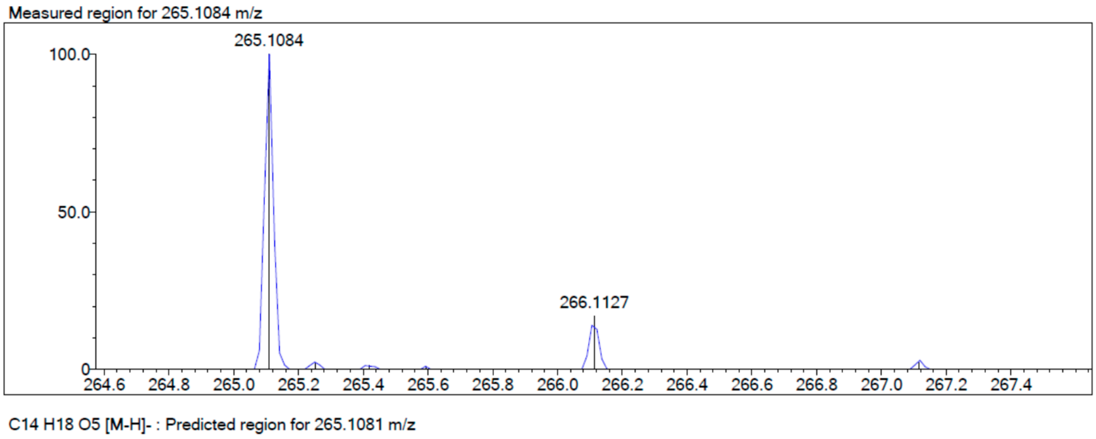

Figure S23. HR ESIMS of **9.**

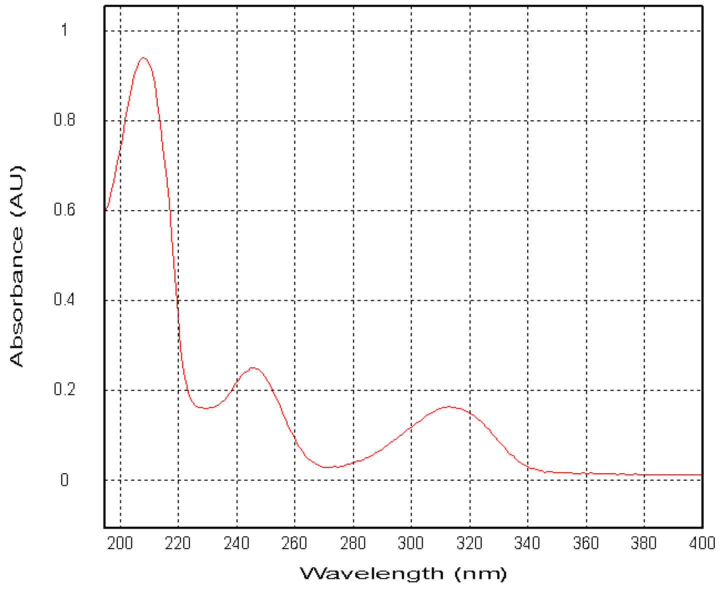

Figure S24. UV spectrum of **9.**

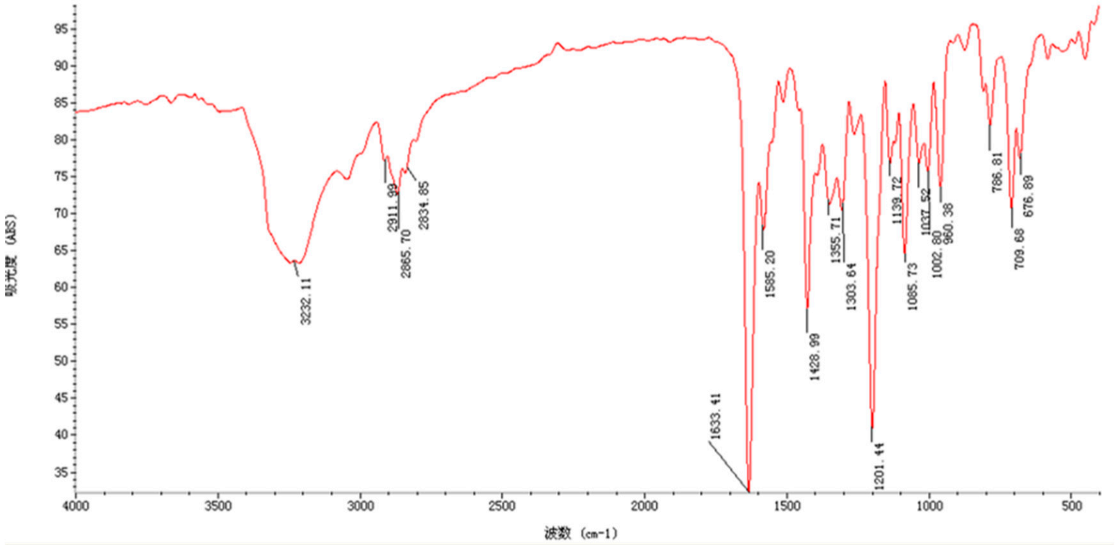

Figure S25. IR spectrum of **9.**

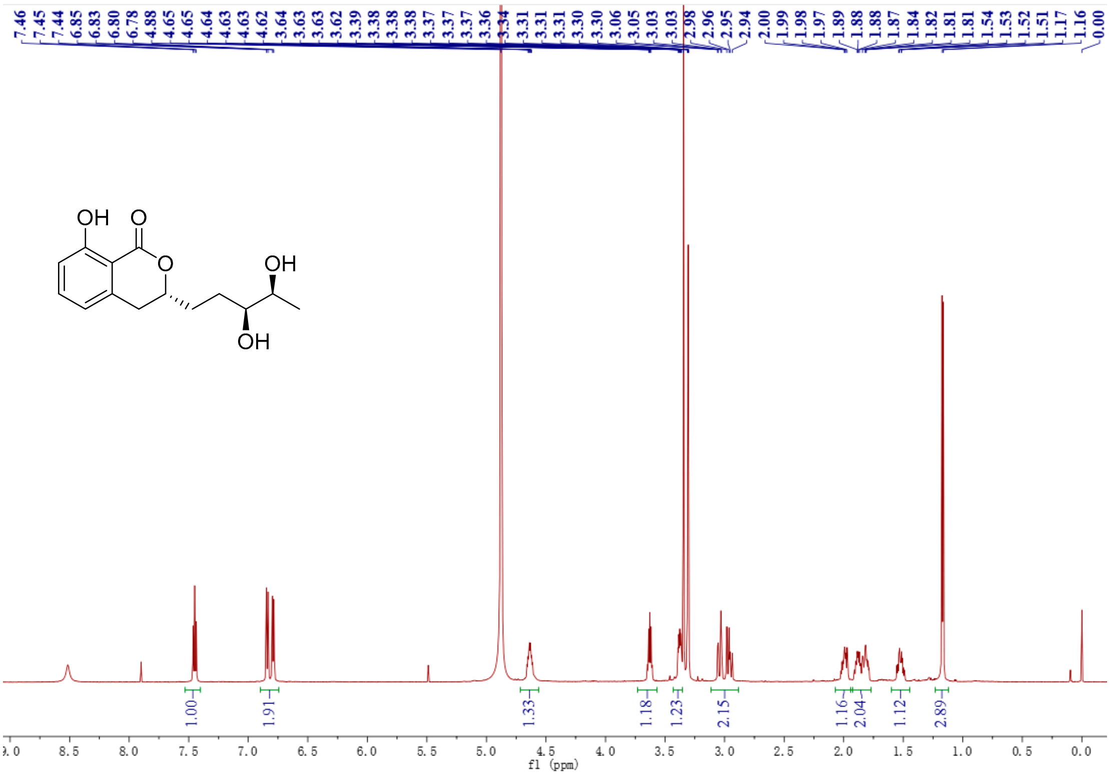

Figure S26. ^{1}H NMR spectrum of **9** (600 MHz, CD_3OD).

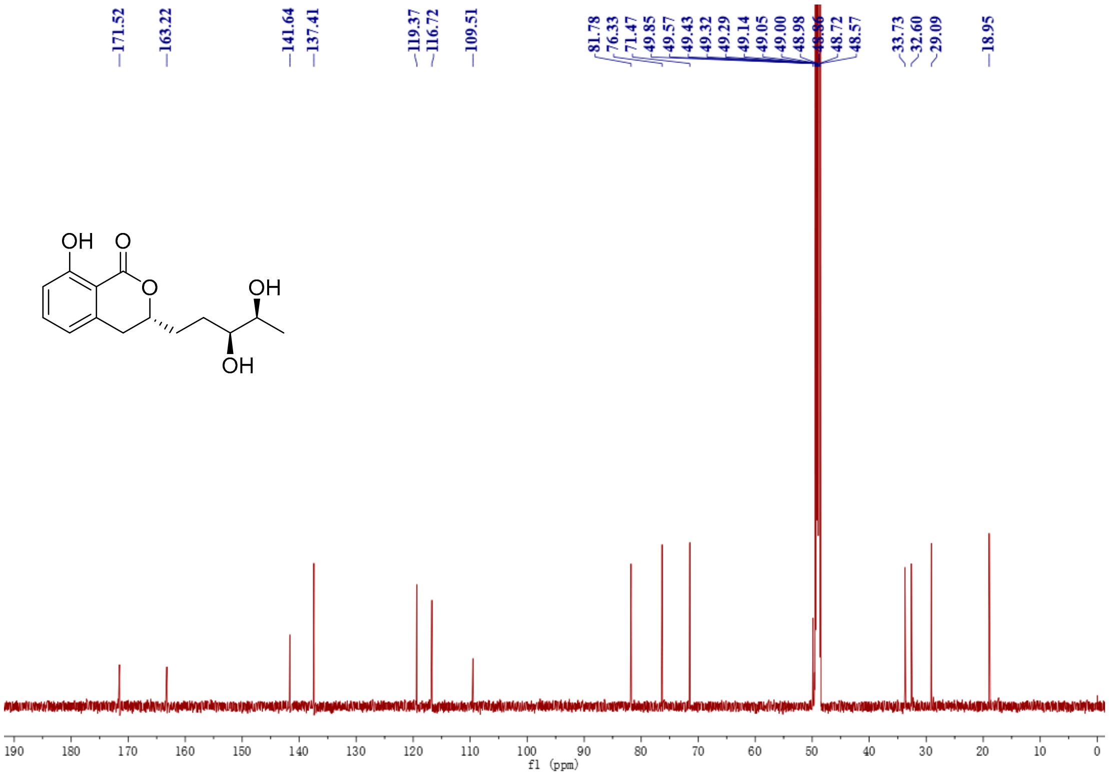

Figure S27. ^{13}C NMR spectrum of **9** (150 MHz, CD_3OD).

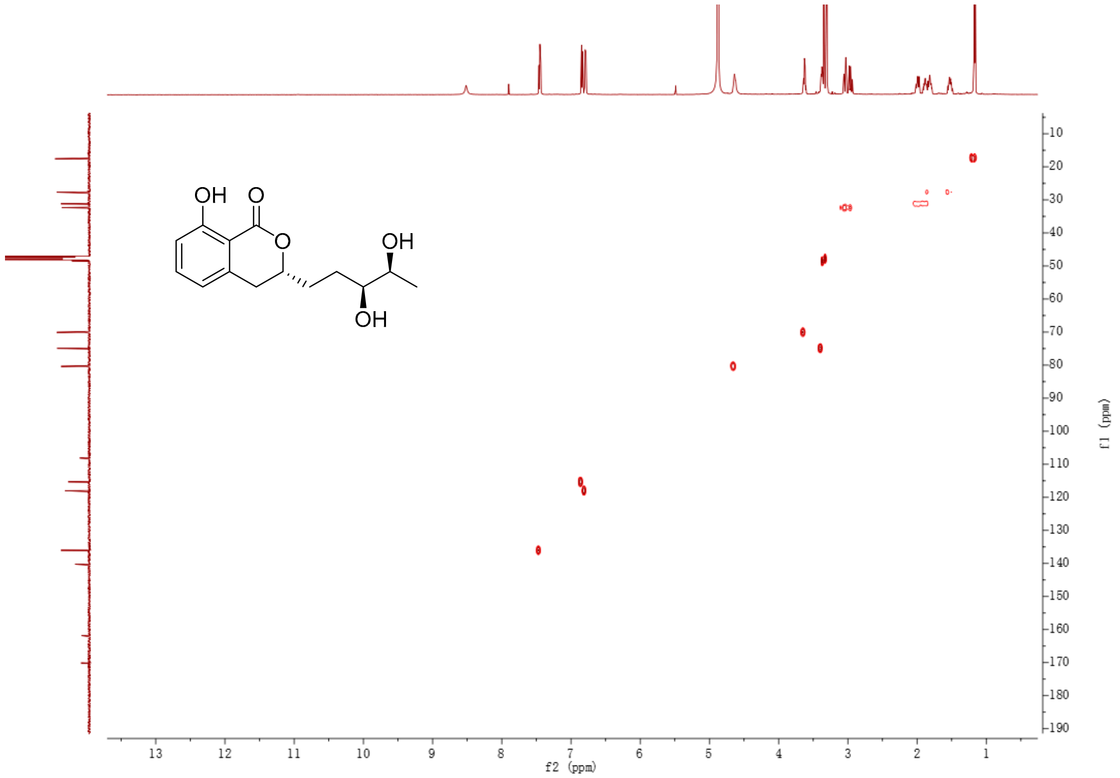

Figure S28. HSQC spectrum of 9 (600 MHz, CD_3OD).

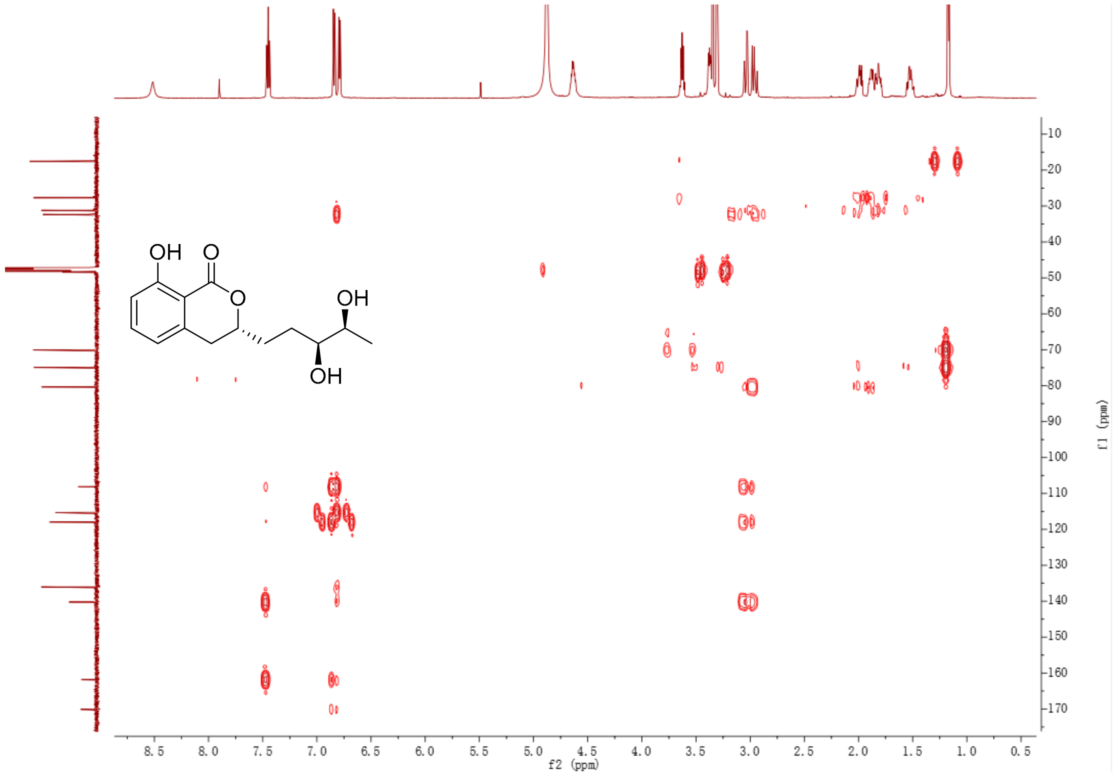

Figure S29. HMBC spectrum of **9** (600 MHz, CD_3OD).

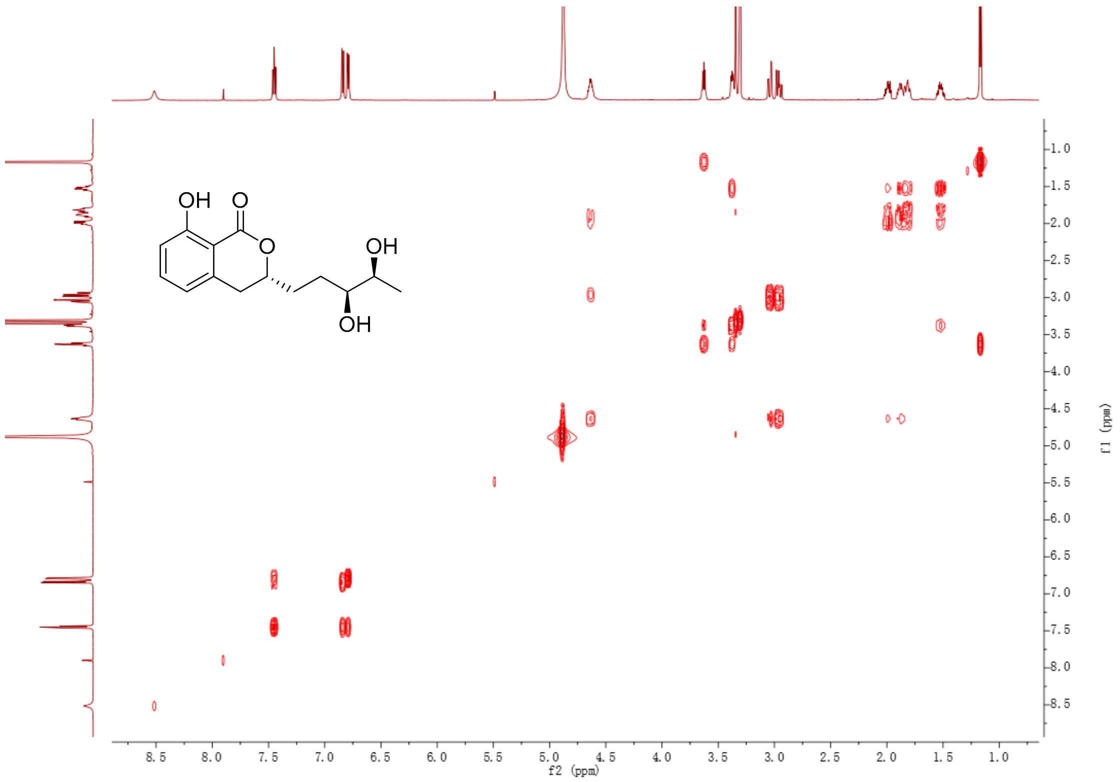

Figure S30. ^{1}H-^{1}H COSY spectrum of **9** (600 MHz, CD_3OD).

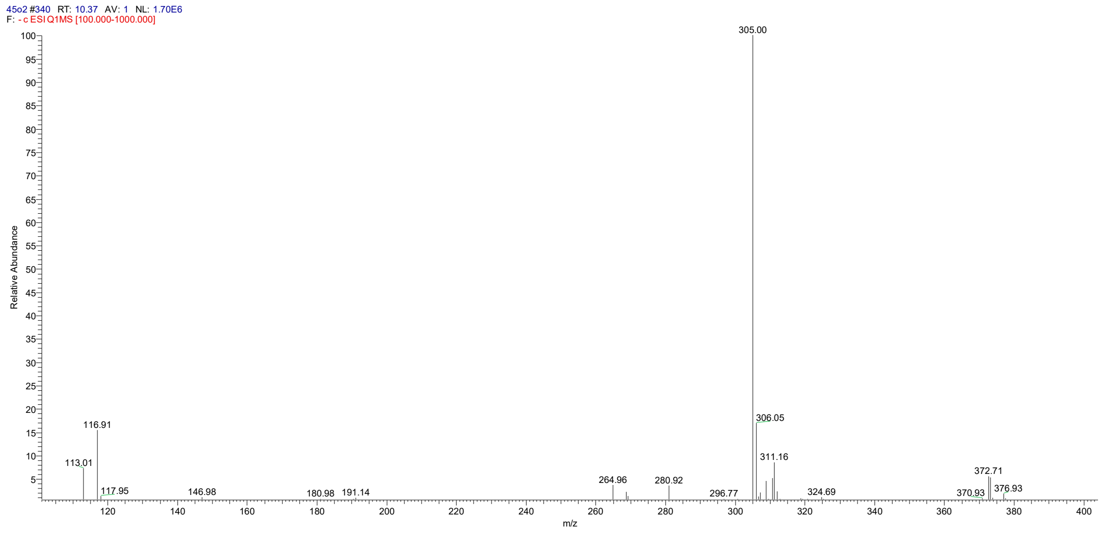

Figure S31. ESI-MS of **9a** (neg.).

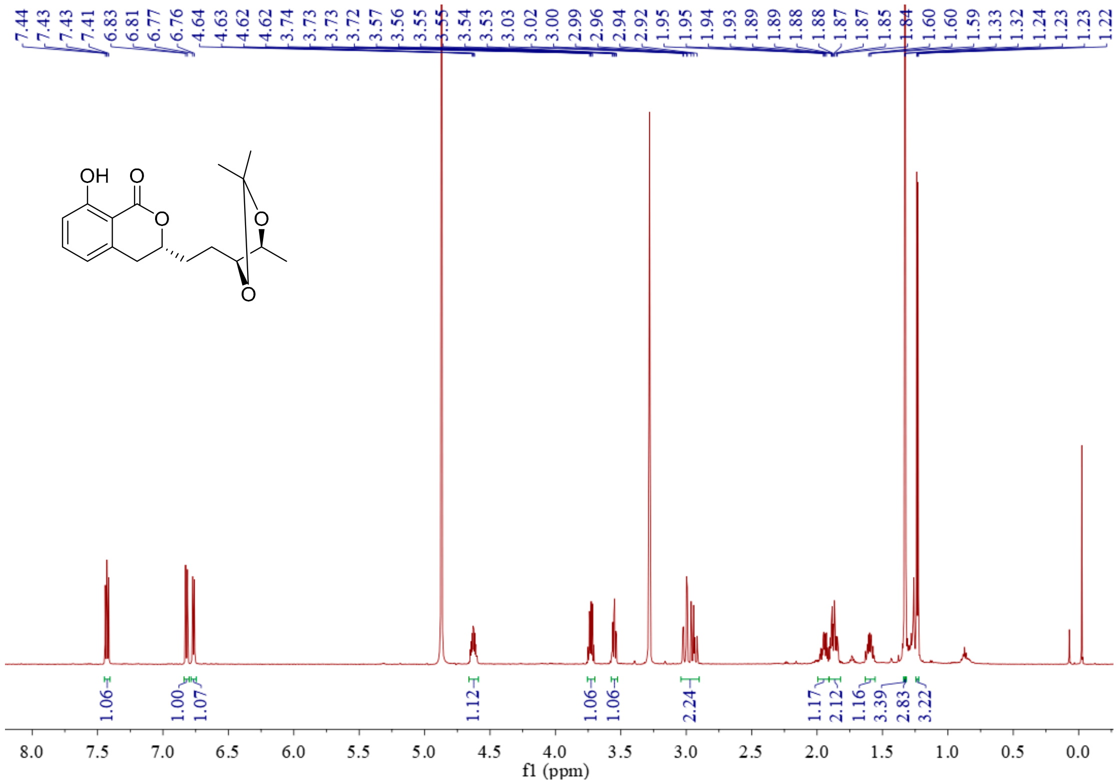

Figure S32. ^1H NMR spectrum of **9a** (600 MHz, CD_3OD).

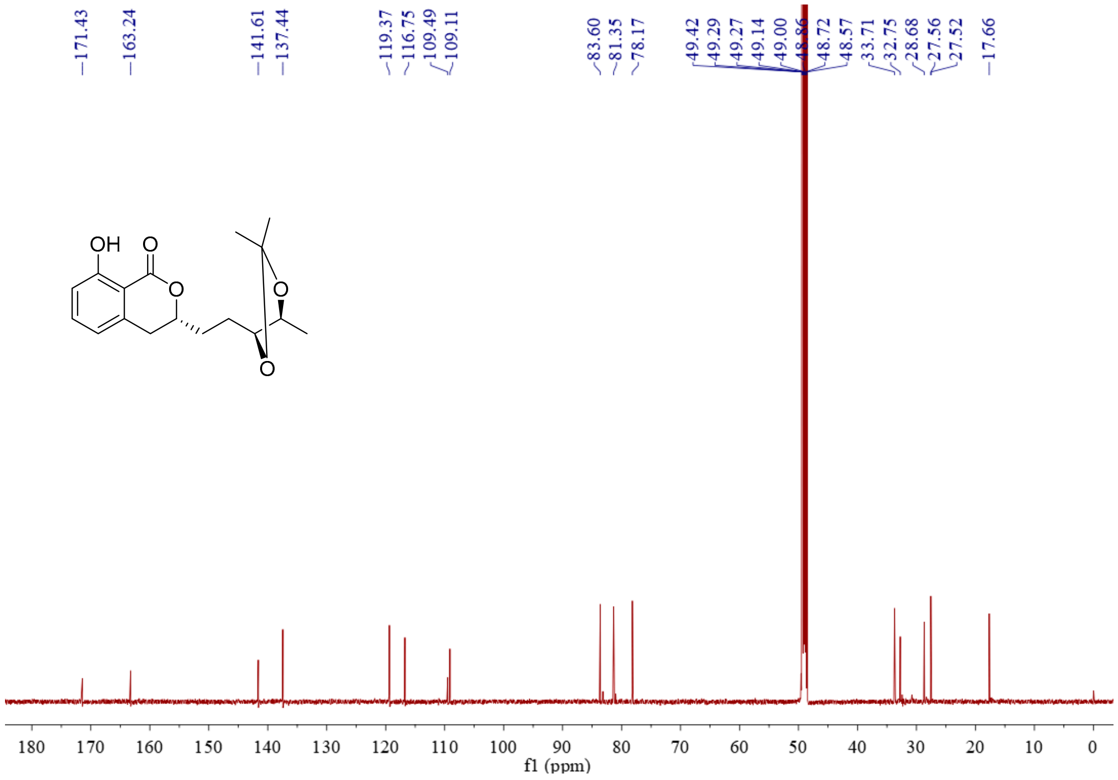

Figure S33. ^{13}C NMR spectrum of **9a** (150 MHz, CD_3OD).

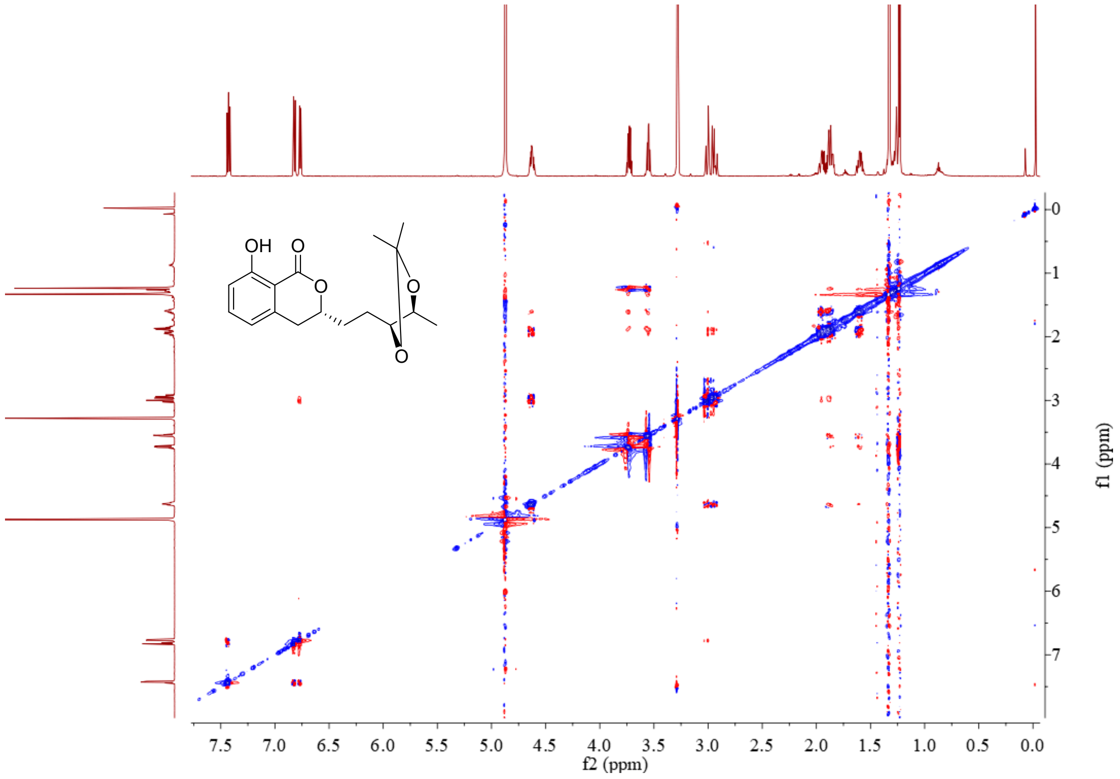

Figure S34. NOESY spectrum of **9a** (600 MHz, CD_3OD).

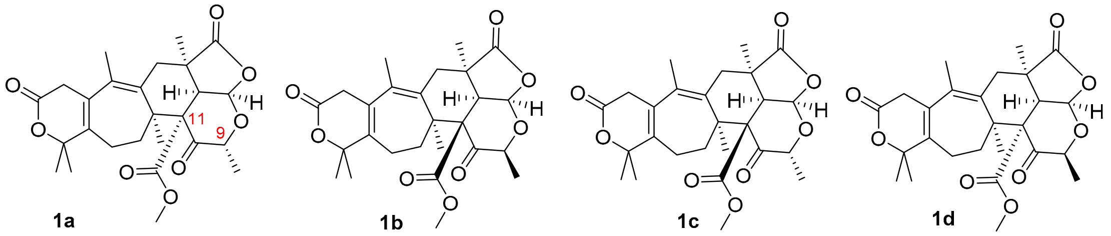

Figure S35. Isomers (at C_9 and C_{11}) of compound **1.**

Table S1. Parameters of the calculated chemical shifts (C data) of compound **1a-1d**.

Isomers	1a	1b	1c	1d
DP4+ (C data)	100 %	0 %	0 %	0 %
CMAD	1.96	2.84	2.74	2.10
CLAD	3.8	13.12	6.52	4.18
R2	0.9985	0.9953	0.9968	0.9982
RMSD	2.2929	4.1136	3.3707	2.5287

References

1. Jiang, X.-Z.; Yu, Z.-D.; Ruan, Y.-M.; Wang, L. Three new species of *Talaromyces* sect. *Talaromyces* discovered from soil in China. *Sci. Rep.*-UK **2018**, *8*, 4932.
2. Xu, G.-B.; He, G.; Bai, H.-H.; Yang, T.; Zhang, G.-L.; Wu, L.-W.; Li, G.-Y. Indole Alkaloids from *Chaetomium globosum*. *J. Nat. Prod.* **2015**, *78*, 1479-1485.
